# Defining the role of *Pseudomonas aeruginosa* PilY1 in signaling and virulence

**DOI:** 10.1128/jb.00200-25

**Published:** 2026-02-09

**Authors:** Christopher L. Pritchett, F. H. Damron, M. Barbier

**Affiliations:** 1 College of Public Health, East Tennessee State University 144478, Johnson City, Tennessee, USA; 2 Department of Microbiology, Immunology, and Cell Biology, West Virginia University 5631, Morgantown, West Virginia, USA; Dartmouth College Geisel School of Medicine, Hanover, New Hampshire, USA

**Keywords:** *Pseudomonas*, pathogenesis, two-component system, gene regulation

## Abstract

*Pseudomonas aeruginosa* (Pa) is an important opportunistic pathogen that has many virulence factors expressed in a coordinated manner to cause infection. The PilY1 protein is a component of the type IV pili (T4P) but has additional signaling functions outside of the pilus. A major function of PilY1 is to prevent the AlgZ/R two-component system from functioning. However, the complete effects of PilY1 signaling through the control of the AlgZ/R or other regulatory systems are not well understood. In this study, we determine new genes controlled by the AlgZ/R system that are impacted by PilY1. We discovered that PilY1 impacts cAMP by preventing the AlgZ/R system from activating the major adenylate cyclase, *cyaB*. PilY1 also prevents the AlgZ/R system from activating itself and a putative c-di-GMP phosphodiesterase, *PA4781*. Furthermore, PilY1 functions in different *P. aeruginosa* strains, including mucoid strains. Surprisingly, bacterial survival in the lung, liver, and blood did not require PilY1. Overall, these findings suggest that PilY1 is critical for preventing AlgZ/R activity to prevent improper second messenger production. This work identifies new AlgZ/R targets and implicates the AlgZ/R system in the control of cAMP in *P. aeruginosa*. This work identifies a possible way to use PilY1 to prevent the expression of the major adenylate cyclase using PilY1 which could be used to impact *P. aeruginosa* virulence.

**IMPORTANCE:**

Understanding PilY1 signaling is important for elucidating how *P. aeruginosa* adapts to different environments. A major function for PilY1 is to interfere with the AlgZ/R two-component system. Here, we use transcriptomics to determine genes PilY1 affects through the AlgZ/R system. We identified new AlgZ/R targets and established a mechanism for impacting cAMP levels. Our findings further our understanding of PilY1 and the AlgZ/R system and suggest a possible way that PilY1 might be used to prevent cAMP from increasing in *P. aeruginosa*.

## INTRODUCTION

*Pseudomonas aeruginosa* is a human opportunistic pathogen capable of causing fatal infections in immunocompromised individuals such as those undergoing chemotherapy, those suffering from severe burn wounds, chronic obstructive pulmonary disease (COPD), or cystic fibrosis (CF) (1–3). Intrinsic and acquired drug resistance has made *P. aeruginosa* a microbe to fear in the clinical setting (4, 5). In the disease process, the adherence to host tissues is a prerequisite for colonization, and the type IV pili (T4P) play a dominant role in adhesion (6–9). Upon inhalation or aspiration, *P. aeruginosa* uses the T4P to colonize the respiratory tract by allowing movement along epithelial cells and forming microcolonies to initiate infection (9). A component of the T4P, PilY1, also has an additional role outside of the T4P. Recent work has discovered that PilY1 is also important in signaling when the bacteria have encountered a surface (10–12). Understanding the molecular events that transpire during these initial events can inform the development of new treatment modalities to combat *P. aeruginosa* infection.

The T4P are made up of the major pilin subunit PilA and the minor pilins FimU-PilVWXE (13–16). PilY1 is the tip adhesin of the T4P and functions in twitching motility, adherence, and mechanosensing (7, 12, 17–19). The transcriptional regulators Vfr and the AlgZ/R two-component system control expression of the *fimU* operon, which is a polycistronic operon that encodes *pilY1* (20–23). The AlgZ/R system is comprised of the membrane histidine kinase AlgZ and the response regulator AlgR which activate transcription of numerous genes (24). AlgR can function in both the unphosphorylated and phosphorylated form (25–28). Once made, PilY1 prevents activation of the AlgZ/R system resulting in a negative feedback loop (12, 29). The *fimU* operon is the only AlgZ/R target known to be affected by PilY1. As the AlgZ/R system controls numerous genes, we hypothesized that PilY1 affects additional genes through the AlgZ/R system other than the *fimU* operon (which includes *pilY1*), but this has not been investigated.

PilY1 has also been found in the inner membrane as well as outside the cell as part of the pilus (7, 30, 31) suggesting that PilY1 has functions outside the T4P. Indeed, another study found PilY1 was important in controlling secondary metabolites, cell density, and persistence in a non-motile strain (32). This study found that PilY1 was functional even in chronic-infecting strains of *P. aeruginosa*. Strains found in chronic infections, such as cystic fibrosis (CF), can acquire mutations in *mucA* causing an overproduction of the polysaccharide alginate (33–35). Therefore, PilY1 may be important in both acute and chronic infections. However, whether PilY1 signaling is similar in different strains has not been determined.

PilY1 signaling impacts second messengers with one known mechanism for regulating c-di-GMP. PilY1 senses surfaces upregulating c-di-GMP levels to increase biofilm formation (10, 12, 36). In contrast, PilY1 was shown to be important for upregulating cAMP upon surface binding (11, 12). However, no mechanism for how PilY1 controls cAMP is known. Other studies have demonstrated that cAMP and c-di-GMP are inversely correlated (37, 38), so how PilY1 affects both c-di-GMP and cAMP has not been clarified. If PilY1 were to increase cAMP levels, then this could explain decreased virulence of *pilY1* mutants in various pathogenesis models (29, 32) due to decreased virulence gene expression. The transcriptional activator Vfr binds cAMP leading to increased production of several virulence factors, including the type III secretion system (11, 22). PilY1 involvement in cAMP has not been closely examined, and this would greatly benefit our understanding of the interactions of various regulatory systems.

While PilY1 is important for signaling, the full extent of the downstream impacts of PilY1 is not well understood. Using RNAseq and other genetic and biochemical methods, we identify other targets in the PilY1 signal cascade. We determined that many of the upregulated genes were due to the AlgZ/R two-component system adding to the AlgZ/R regulon. This work furthers our understanding of the PilY1 signaling cascade and the AlgZ/R system.

## MATERIALS AND METHODS

### Bacterial strains, plasmids, and growth conditions

Bacterial strains and plasmids used in this study are listed in Table S1. *P. aeruginosa* was grown at 37°C in Luria Bertani (LB) broth supplemented with 50 mM MOPS or LB agar for routine growth. Transcriptional fusion assays were performed in LB Mops (50 mM), quorum sensing media (QSM, 22 mM KH_2_PO_4_, 22 mM Na_2_HPO_4_, 85 mM NaCl, 10% tryptone, 1 mM MgSO_4_, 0.1 mM CaCl_2_). Media used for *P. aeruginosa* was supplemented with tetracycline (180 μg/mL), gentamicin (250 μg/mL), carbenicillin (300 μg/mL), or trimethoprim (500 μg/mL) as needed. *E. coli* was cultivated at 37°C in LB and supplemented when necessary with ampicillin (100 μg/mL), kanamycin (35 μg/mL), or chloramphenicol 34 μg/mL. Yeast-tryptone media (YT, 1% tryptone, 0.5% yeast extract) was used for allelic exchange experiments and was supplemented with the appropriate antibiotics or with 10% sucrose. In the case of complementation, spectinomycin (25 μg/mL) was used in combination with trimethoprim (500 μg/mL).

### Mutant strain construction

PCR-generated fragments were amplified using Q5 polymerase (New England Biolabs) and cloned directionally into either pEX18Tc or pEX18Gm (39) after performing cross-over PCR (40). Mutant constructs were introduced into PAO1 or other strains via tri-parental mating using the helper strain pRK2013 (41). Single crossover mutants were selected on the appropriate selective media, and merodiploids were grown without selection to select for a second crossover event. Mutants were plated on YT 10% sucrose and then patch-plated on selective media and PIA. Colonies not growing on selective media were screened via PCR for the appropriate mutation. Complementation was accomplished by PCR of the corresponding wild-type gene using Q5 (NEB) and cloning into the integrating vector pTJ1 (42). Sequencing was used to confirm all constructs and mutant strains. Strains are listed in Table S1, and primers used for making strains are listed in Table S2.

### RNA seq

Total RNA was isolated from the selected strains using the RNA SNAP procedure (43) and DNase-treated. The RNA was purified using RNeasy kit (Qiagen), quantified using Nanodrop ND-1000 (Nanodrop), and assessed for RNA integrity using Agilent BioAnalyzer RNA Pico chip (Agilent). A total of 50 μg from each sample was sent for RNAseq analysis (Admera Health). All samples were submitted to rRNA depletion (RiboMinus Transcriptome Isolation Kit, ThermoFisher) and reassessed for RNA integrity. rRNA-depleted mRNA samples were then fragmented and prepared into libraries using Illumina TruSeq RNA library prep kit v2 (Illumina). The libraries were then sequenced on an Illumina HiSeq 2 × 150 bp reads with a total of 10^6^ reads per sample and 3 replicates per experimental condition. Sequencing data were deposited to the GEO website and are available under the reference number GSE278651. RNA-seq reads were analyzed using the software CLC Genomics Workbench (Qiagen). The *P. aeruginosa* PAO1 genome and annotations were downloaded from the Pseudomonas Genome Database, and reads were mapped using the following settings: mismatch cost = 2, insertion cost = 3, deletion cost = 3, length fraction = 0.8, similarity fraction = 0.8. RPKM values were generated using default parameters for CLC Genomics. Fold changes in gene expression and statistical analyses were performed using an Extraction of Differential Gene Expression (EDGE) test with the Bonferroni correction.

### Transcriptional fusion analysis

Upstream DNA fragments containing promoter regions were generated by using primers listed in Table S2 in conjunction with Q5 polymerase (New England Biolabs, Ipswich, MA). PAO1 genomic DNA was used as template. PCR products were cloned into pMiniT (NEB) and then sub-cloned into miniCTXlacZ or miniCTXlux using the restriction enzymes *Hind*III*/Bam*HI, *Hind*III/*Eco*RI, or *Kpn*I/*Bam*HI (NEB). PCR products were purified, cut with restriction enzymes, and inserted into the EcoRI/BamHI sites of miniCTXlacZ using T4 DNA ligase (NEB). Strains were selected for tetracycline resistance and then conjugated with pFLP2 to remove vector sequences in the case of *lacZ* fusions (39). Strains were selected for carbenicillin resistance, grown overnight without selection, and plated on YT media with 10% sucrose to select for the loss of pFLP2. Individual colonies were patch-plated onto VBMM CB300 and PIA to ensure the loss of pFLP2. To confirm the presence of the fusion constructs, PCR was performed using the forward primer used to construct the fusion and the reverse primer, lacZRforTF (Table S2). β-Galactosidase activity was determined by incubating cell extracts with ONPG (4 mg/mL) as per Miller (44). A strain carrying the empty vector, miniCTXlacZ, was also conjugated into PAO1 and assayed, and this background (28 Miller Units) was subtracted from all transcriptional fusions. All mucoid strains were confirmed mucoid at the end of each experiment by plating on PIA plates to ensure all colonies were mucoid. All assays were performed in buffered LB (LB + 50 mM MOPS). At least three biological replicates were reproduced for all assays.

For *lux* fusions, promoter fragments were cloned into miniCTXlux and conjugated into *P. aeruginosa* strains (45). The vector sequences were not removed. White-walled plates in a Synergy HTX (Bio Tek) were used for assays. Strains were grown overnight and diluted 1:50 in LB MOPS (50 mM), and 200 μL was used to inoculate white-walled plates containing LB MOPS (50 mM). Strains were measured every 30 min for 5–10 h using a Synergy HTX or a Synergy LX plate reader. For complementation studies, arabinose (0.1%–1%) was added to media before diluting strains grown to logarithmic phase. Data were analyzed using GraphPad Prism. All experiments were repeated at least three times in triplicate for each strain. Data are reported as relative luminescence by dividing luminescence readings by the OD_600_ for luminescence assays and in Miller Units for β-galactosidase assays. Area under the curve analysis was used to compare strains using GraphPad Prism. Error bars indicate the standard error of the mean.

### AlgR purification and antibody production

The *algR* gene was cloned into the expression vector pMALc6t expression vector (NEB). AlgR was expressed as a fusion with maltose-binding protein (MBP). The fusion protein was batch purified using agarose resin and cleaved using TEV protease, and the TEV and MBP were removed using Ni-affinity chromatography. AlgR was dialyzed using a slidalyzer (ThermoFisher) and Storage Buffer (20% glycerol, 20 mM Tris pH 7.5, 5 mM MgCl_2_, and 1 mM DTT) overnight at 22°C. The purity of AlgR was visually determined in a Coomassie stained 4%–12% gradient electrophoresis gel (SDS-PAGE), and AlgR was confirmed by western blots using AlgR-specific sera. Antibodies were produced to AlgR by ProSci (Poway, CA).

### Electrophoretic mobility shift assays

Gel mobility shift assays are described previously with some modifications (46, 47). PCR fragments were generated using biotinylated primers (see Table S2) and gel purified using the Monarch Gel Extraction kit (NEB). Binding reactions were carried out using purified AlgR. The DNA (50–200 ng) probes were mixed with AlgR protein containing 20 mM Tris-HCl (pH 8.0), 0.5 mM dithiothreitol, 20 mM KCl, 0.5 mM MgCl_2_, 2 mM EDTA, and 5% glycerol. The nonspecific competitor poly (dI-dC) was added at 10 μg/mL for all gel shift reactions. After incubation for 20 min at room temperature (25°C), the samples were separated by electrophoresis on a 6% native polyacrylamide gel with 0.375 × TBE used as running buffer for approximately 1.5 h at 100 V. Purified AlgR was incubated at increasing concentrations to determine suitable concentrations to be used. The gel shifts were developed using the chemiluminescent detection kit (Life Technologies) and visualized using a Bio-Rad chemi-doc. Gel shifts were repeated at least three times, and a representative gel shift is shown.

### Site-directed mutagenesis

Primers are listed in Table S1. The primers were phosphorylated and used in site-directed mutagenesis as per the manufacturer’s instruction using Q5 (NEB). Constructs were analyzed by restriction enzyme analysis and sequencing. Mutant strains were constructed using homologous recombination as described above and were checked using PCR and primer pairs in Table S1. Site-directed mutants had an *Eco*RI site engineered to allow easier detection of the promoter mutation. Additional PCR amplicons were sequenced to confirm the mutation in each strain using the same primers. Further confirmation of mutants was done using phenotypic or biochemical assays.

### Western blot analysis

The bacteria were collected by centrifugation and resuspended in 50 mM Tris-HCl pH 7.5 and lysed using sonication or B-PER (Fisher Scientific). Total protein concentrations were quantified by the Bradford protein assay (Bio-Rad). Cell extracts (10 μg) were separated by SDS-PAGE on 10% polyacrylamide gels and transferred to a polyvinylidene difluoride membrane (GE Osmonics). The membranes were probed using a 1:10,000 dilution of anti-AlgR rabbit polyclonal antibody followed by a 1:20,000 dilution of horseradish peroxidase-conjugated goat anti-rabbit monoclonal antibody (48), and the signal was detected using chemiluminescence (Bio-Rad). The anti-AlgR polyclonal antibody was pre-absorbed with cell extract from the *∆algR* strain to remove other non-specific antibodies.

Westerns were developed using ECL reagent (ThermoScientific) and imaged using a Chemi-Doc (Bio-Rad).

### Phenotypic assays

Pyocyanin assays were performed as described in reference 49. Briefly, overnight cultures in QSM media were pelleted and the supernatant filtered through a 0.2 μm filter. Three milliliters of supernatant was extracted twice with 1 mL of chloroform and vortexed vigorously. The organic layer was removed, and 1 mL of 0.2N HCl was added. The OD_520_ was taken, and the samples were normalized to the OD_600_ of the culture. Blanking of the spectrophotometer was done using 0.2 N HCl. Biofilm assays were performed as described previously (50).

### Competition experiments

All animal protocols were approved by the Institutional Animal Care and Use Committee at East Tennessee State University (P220301) following the guidelines of the Office of Laboratory Animal Welfare. Overnight cultures were diluted 1:100 in LB and grown for 3–5 h, pelleted, and washed two times with PBS. The OD_600_ of each strain was adjusted to an OD_600_= 0.2. Further dilution with PBS was done to achieve the final dose of 1 × 10^7^ CFUs. Mutant and wild-type strains were mixed 1:1, and the input was determined by plating on PIA and PIA gent. Groups of three to five 12–16 week CD-1 mice (Envigo) were infected by oropharyngeal aspiration after anesthetization with 2.5% isoflurane with *P. aeruginosa* at doses of 1 × 10^7^ CFUs in 40 μL. Sixteen hours after infection, mice were euthanized, and blood, lungs, livers, and spleens were collected. Organs were homogenized in 1 mL of PBS, and all tissues were serially diluted and 100 μl plated in duplicate on both PIA and PIA Gent. Competitive indices were determined by dividing the output ratio by the input ratio.

### Statistical analysis

Statistics were performed using GraphPad/Prism 6.0 (GraphPad software, La Jolla, CA). Transcriptional fusions and pyocyanin levels were compared using a one-way analysis of variance (ANOVA) with a Tukey posttest. Image J software was used for western blot analysis.

A student’ *t*-test was used for beta-galactosidase assays for multiple strains comparing the mutant to the wild-type. All kinetic assays using *lux* reporter and EMSA’s were performed at least three times. Kinetic *lux* assays, beta-galactosidase, and pyocyanin assays were performed in duplicate or triplicate with three biological replicates.

## RESULTS

### PilY1 affects cAMP levels differently than other pilin components

**Fig 1:**
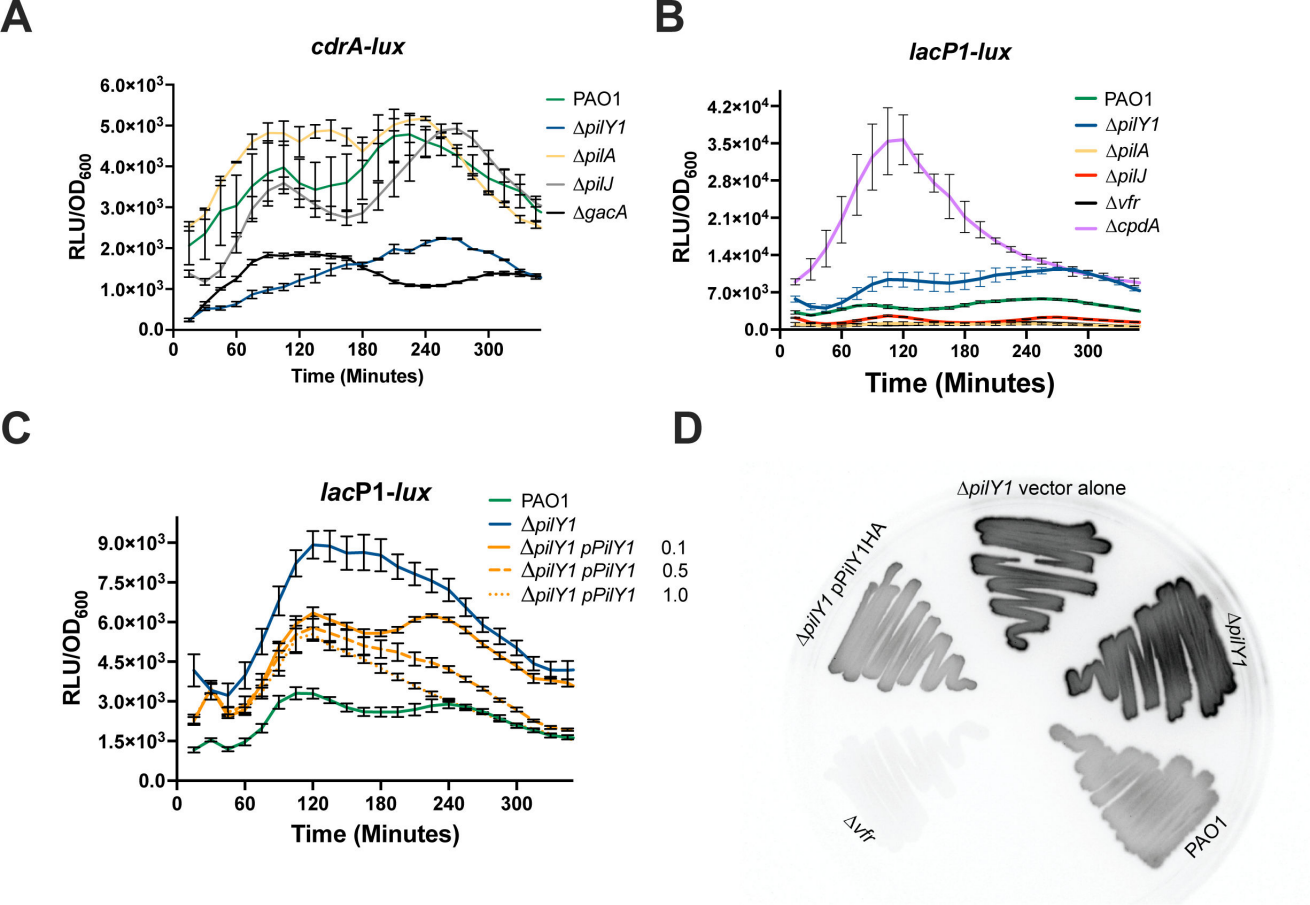
PilY1 affects both cAMP and c-di-GMP in *P. aeruginosa*. Kinetic analysis of c-di-GMP and cAMP levels. (**A**) *cdrA* promoter activity representative of c-di-GMP levels. (**B**) *lac*P1-*lux* reporter activity representative of cAMP levels. (**C**) Complementation of ∆*pilY1* reduces cAMP. (**D**) Luminescence of strains containing the *lac*P1-*lux* reporter on solid media after 16 h. Assays represent three biological replicates done in triplicate, and measurements were taken every 15 min for 5 h. Complementation studies were with L-arabinose (0%–1%). An uninduced control was indistinguishable from the *∆pilY1* strain and was left off the graph for simplicity.

One consequence of mechanosensing is the increase in cyclic di-GMP (10, 19). Notably, cAMP levels inhibit c-di-GMP levels in *P. aeruginosa* (37, 38, 51). This led us to speculate that PilY1 might impact cAMP levels in *P. aeruginosa,* and this could explain decreased c-di-GMP in the *pilY1* mutant. Transcriptional *lux* reporters, encoding the *luxCDABE* genes responsible for luminescence, were constructed to monitor cAMP and c-di-GMP production over time. The advantage of using *lux* reporters is this allows real-time evaluation of expression in live cells without the need for lysing cells and adding exogenous substrates. We integrated the second messenger sensors into the *P. aeruginosa* chromosome to analyze the transcriptional fusions in single copy (45, 52). A *cdrA* transcriptional fusion was constructed and used as a sensor for c-di-GMP as this promoter has been shown to correlate with c-di-GMP levels (53). The strains were grown with shaking in triplicate in buffered LB (LB + 50 mM MOPS) and monitored for luminescence and optical density over time (300 min) at 37°C. The area under the curve was determined for these strains and demonstrated a significant decrease in relative luminescence for *∆pilY1* compared to the wild-type strain (*P* < 0.0001, Table S3). A previous study used a *lux* reporter and also showed a decrease in luminescence in *∆pilY1* (29); however, no statistical analysis was performed. A ∆*gacA* strain was used as a negative control (54), supporting the use of this construct to evaluate c-di-GMP levels. The *cdrA* reporter was also tested in other pilin mutants that are deficient in components that affect cAMP levels and T4P biogenesis (10). A ∆*pilA* and ∆*pilJ* had luminescence like the wild-type strain (Fig. 1A). Neither *∆pilA* nor *∆pilJ* were significantly different from PAO1 (Table S3). These data suggest that our *cdrA* reporter can also be used to assess c-di-GMP levels in *P. aeruginosa*.

The *∆pilY1* mutant was complemented by incorporating a C-terminal influenza hemagglutinin-tagged (HA-tag) version of the wild-type *pilY1* under the control of an arabinose-inducible promoter using an integrating vector to provide single copy complementation (42). This construct was fully functional as determined by complementing twitching motility and biofilm formation (Fig. S1). When ∆*pilY1* was complemented, there was a delay in the response, but increasing arabinose concentration restored *cdrA* reporter activity (Fig. S2). In fact, increasing arabinose to 0.5% and 1.0% resulted in increased *cdrA* reporter activity after 180 min suggesting that increasing the amount of PilY1 leads to increased cyclic di-GMP (Fig. S2). This further supports the importance of PilY1 in controlling c-di-GMP levels.

As cAMP and c-di-GMP levels are often inversely related (38), we also tested a cAMP biosensor to determine if cAMP levels were affected by the same mutations. The *lacP*1 promoter previously shown to correlate with cAMP levels (12, 37, 55, 56) was fused to the *lux* operon (Fig. 1B). A ∆*vfr* mutant was used as a negative control as this strain is not responsive to increased cAMP levels (55). We also tested the cAMP reporter in a ∆*cpdA* mutant as a positive control (57), which had the highest luminescence. A ∆*pilY1* mutant had modestly increased cAMP levels compared to PAO1 using the *lac*P1-lux reporter in a liquid assay, and this was significant when comparing the area under the curve for these two strains (*P* < 0.0001, Table S3) (Fig. 1B). Previous work indicated that both PilA and PilJ were necessary for optimal cAMP levels after surface attachment (10). We would then expect that the cAMP reporter would have decreased activity in these two pilin mutant strains. A ∆*pilA* strain had decreased Lux activity compared to PAO1 and was like the ∆*vfr* strain. Similarly, a ∆*pilJ* strain had slightly decreased luminescence compared to PAO1. Both *∆pilA* and *∆pilJ* were significantly decreased from PAO1 when comparing the area under the curves (Table S3). Therefore, our results are consistent with published results for ∆*pilA* and ∆*pilJ* (11, 12, 55) and indicate our reporters function as expected. This further supports that PilY1 functions differently than other pilin components concerning both c-di-GMP and cAMP. Complementation of the *∆pilY1* strain containing the *lac*P1-*lux* reporter demonstrated decreased cAMP levels even without induction with arabinose (Fig. 1C). Increasing arabinose concentration lowered cAMP reporter activity indicating that PilY1 is responsible for lowering cAMP levels. Complementation using 0.5% or 1% arabinose reduced cAMP to wild-type levels in the complemented strain after 240 min. From these data, we conclude that PilY1 affects cAMP levels in *P. aeruginosa*.

A previous study found that cAMP levels are increased upon growth on surfaces (12). The strains containing the *lac*P1-*lux* reporter were grown on solid media and produced luminescence that was visually striking. The luminescence of the *∆pilY1* was much brighter than the wild-type and the *∆vfr* mutant (Fig. 1D). Complementation with *pilY1* restored the level of luminescence to the wild-type strain (Fig. 1D). These data suggested that PilY1 is important for decreasing cAMP levels and that PilY1 is different from other components such as PilA and PilJ that allow increased cAMP levels (11, 56, 58). This is also consistent with the idea that PilY1 signaling is not due to its presence in the pili as a ∆*pilA* mutant does not express functional pili.

### Transcriptomic analysis reveals genes affected by *pilY1* mutation

**Fig 2:**
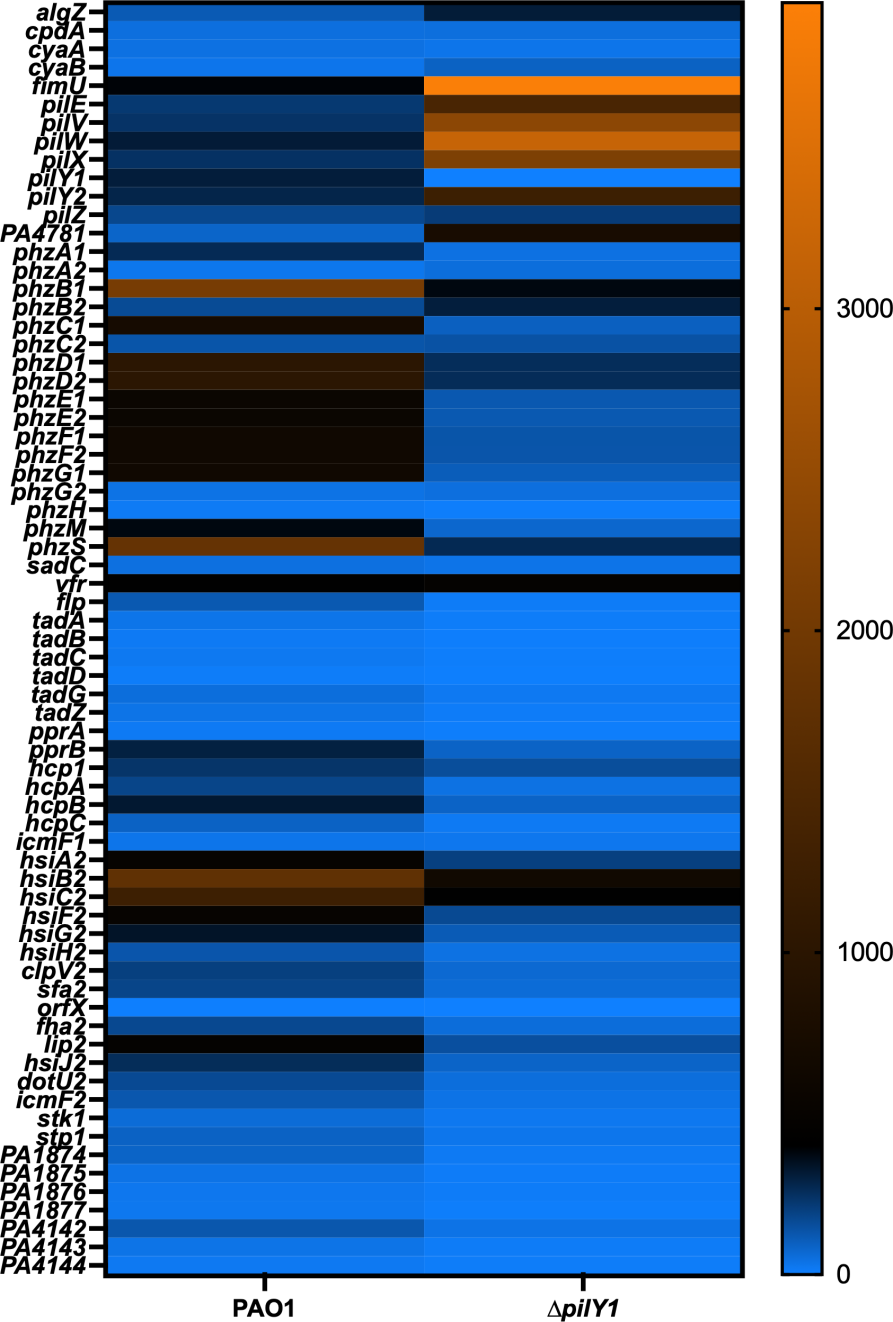
RNAseq analysis defines the PilY1-controlled genes. Heat map comparing differentially regulated genes. The heat map shows the reads per kilobase per million reads (RPKM) for 150 differentially expressed genes between the wild-type PAO1 and the ∆*pilY1* strains.

RNAseq was performed to provide an unbiased view of what other genes PilY1 affects that might explain cAMP regulation as well as determine other transcriptional changes. Comparing the ∆*pilY1* mutant to the wild-type strain PAO1, RNAseq identified 168 genes that were upregulated in the *∆pilY1* strain and 118 that were downregulated (Fig. 2, Supplementary Excel file). PilY1 is known to control pyocyanin (32) and control expression of its own operon (29). As expected, many downregulated genes were involved in pyocyanin production (Fig. 2). There are two operons capable of producing pyocyanin (59). Twelve of the downregulated genes (Supplementary Excel file) were from both operons involved in pyocyanin biosynthesis. The finding of pyocyanin biosynthetic genes validates our use of RNAseq to identify new PilY1 targets.

Other downregulated genes included several secretion systems. Two Type I secretion systems (PA1874–PA1877 and PA4142–4144) that are poorly understood were decreased in the *pilY1* mutant. PA1874–1877 is an ABC transport system that has been shown to be an efflux pump when *P. aeruginosa* is growing as a biofilm (60). Even less is known about the putative secretion cluster PA4142–4144, but it is regulated via quorum sensing (61). However, both type I secretion systems have been increased in expression in burn wounds (62). In addition, the second type VI secretion system (H2-T6SS [63) was also decreased in expression when *pilY1* was deleted.

Interestingly, PilY1 appears important in the expression of type IVb pili. *P. aeruginosa* is one of very few bacteria that expresses both type IVa and type IVb pili (64). Several mRNAs encoded by the *tad* locus and *flp* were severely decreased as well as the *pprAB* system that controls the type IVb structural genes. The type IVb pili are important for DNA transfer (65). Therefore, the RNAseq analysis suggests that there is a hierarchy in the expression of the type IVa and type IVb pili in *P. aeruginosa* with the type IVa pili controlling type IVb expression.

Upregulated genes include *fimU*, *pilX*, *pilW*, *pilE*, and *pilY2* contained within the *fimU* operon that also contains *pilY1* (Fig. 2, Supplementary Excel file). The RNAseq data indicated that PilY1 also impacts the expression of *algZ*, the protein that phosphorylates AlgR (24) and also regulates the *fimU* operon . Numerous unannotated genes were also increased in expression. Interestingly, the major adenylate cyclase, *cyaB* (22), was increased in the *pilY1* mutant. Overall, our RNAseq results identified new genes affected by PilY1 and suggested a possible mechanism for PilY1 control of cAMP levels.

Another goal was to determine other possible explanations for PilY1 control of c-di-GMP or cAMP. There was no decreased expression of any known diguanylate cyclase, but we did find one putative phosphodiesterase that had increased expression: PA4781 (Fig. 2, Supplementary Excel file). An increase in PA4781 could explain the decreased c-di-GMP levels in addition to PilY1 affecting SadC (19, 66).

*P. aeruginosa* controls cAMP levels by two adenylate cyclases, CyaA and CyaB, and one known phosphodiesterase *cpdA* (22, 57). The RNAseq data did not indicate an increase in the main cAMP phosphodiesterase *cpdA*. We confirmed this result using a transcriptional reporter to the *cpdA* promoter and found that *cpdA* expression was slightly reduced (Fig. S3A). However, we did find an increase in the major adenylate cyclase *cyaB* in the RNAseq analysis. Therefore, the RNAseq results suggested that increased cAMP was due to increased *cyaB* expression and not increased expression of the phosphodiesterase that breaks down cAMP.

### The major adenylate cyclase, *cyaB*, is increased in the ∆*pilY1* strain

**Fig 3:**
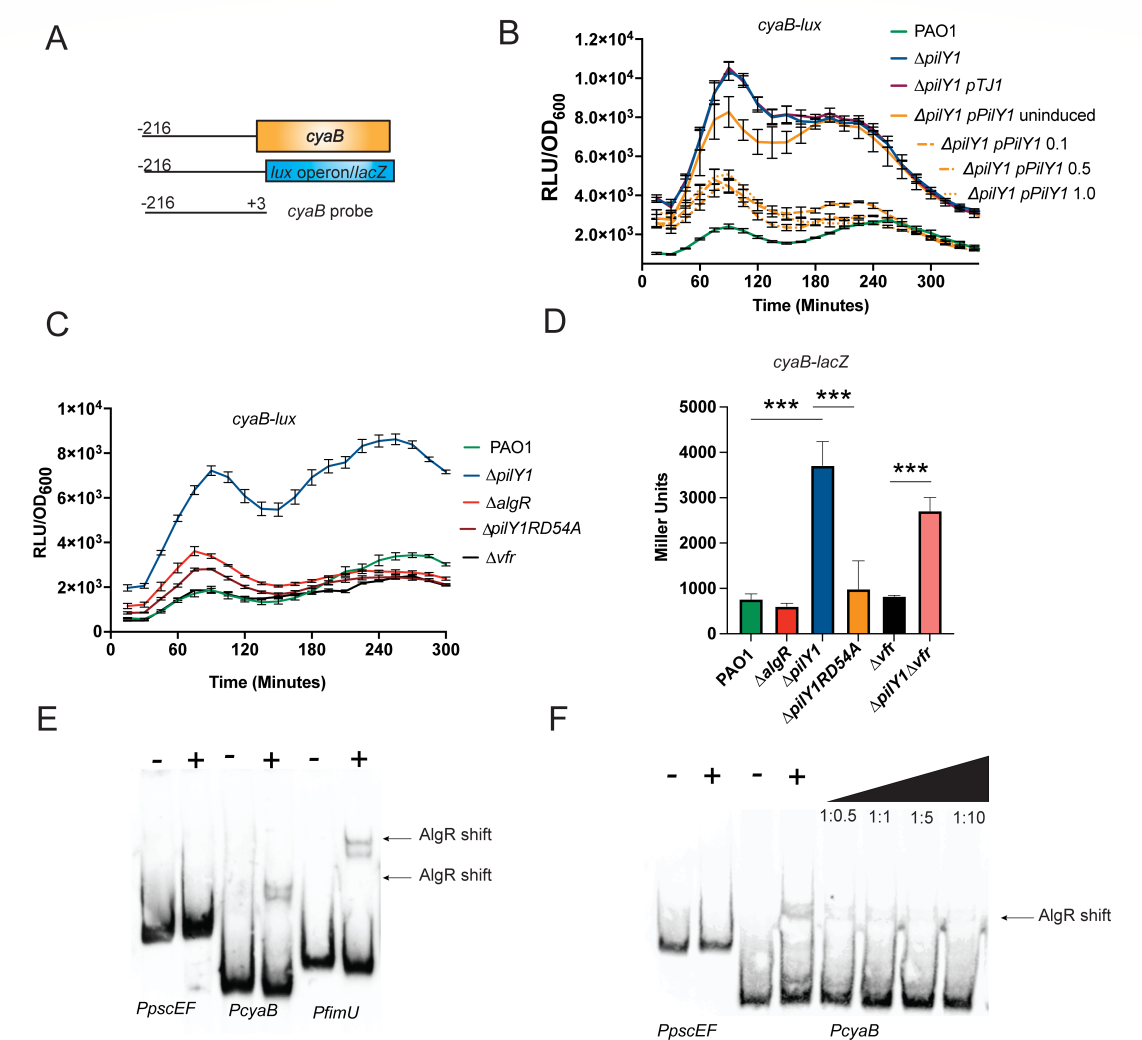
PilY1 controls *cyaB* using the AlgZ/R system. (**A**) Schematic showing the region of the *cyaB* promoter used for constructing transcriptional fusions and making the probe for EMSA analysis. (**B**) A *cyaB-lux* transcriptional fusion has increased activity in the ∆*pilY1* strain, and overexpression of *pilY1* leads to decreased *cyaB* expression. (**C**) The AlgZ/R system is important in regulating *cyaB-lux*. (**D**) Confirmation of AlgZ/R regulation of *cyaB* using a *cyaB-lacZ* transcriptional fusion. (**E**) EMSA analysis of the *cyaB*, *pscEF*, and *fimU* probes using purified AlgR. (**F**) Competition using unlabeled *cyaB* probe demonstrates the specificity of AlgR binding to *cyaB* promoter. Competitions were done using 0.5:1, 1:1, 1:5, and 1:10 ratios of unlabeled to labeled probe, respectively. A one-way ANOVA with Tukey’s post-test was used for statistical significance (***, *P* < 0.0001).

The RNAseq data showed that *cyaB* was increased in the *∆pilY1* mutant. This could explain the slight increase in cAMP detected in the *∆pilY1* strain (Fig. 1B). CyaB provides most of the cAMP in the *P. aeruginosa* cell (22). We constructed a *cyaB-lux* transcriptional fusion to monitor *cyaB* expression kinetically and detected increased reporter activity in the ∆*pilY1* strain (Fig. 3A and B). A comparison of PAO1 and *∆pilY1* containing the *cyaB-lux* fusion was statistically significant (*P* < 0.0001, Table S3). This result confirms the RNAseq data and shows that *cyaB* expression is increased in the *∆pilY1* strain. We complemented the ∆*pilY1* strain containing the *cyaB-lux* fusion (Fig. 3B). As little as 0.1% arabinose decreased expression of *cyaB-lux* in the complemented strain (Fig. 3B). From these data, we conclude that PilY1 does affect the expression of *cyaB*.

To determine if PilY1 affects the expression of other genes encoding proteins that make or degrade cAMP, transcriptional *lux* reporters were constructed of *cyaA* and *cpdA* (Fig. S3). We also tested a ∆*vfr* mutant as Vfr and cAMP are intimately linked and Vfr has been demonstrated to directly activate *cpdA* expression (57). Compared to the wild-type strain, only *cyaB* differed significantly in the expression of the adenylate cyclase genes in the *pilY1* mutant (Fig. 3C; Fig. S3). Interestingly, Vfr did not affect the expression of *cyaB*, but *cyaA* was slightly increased in a *vfr* mutant at later timepoints (Fig. S3B). There was a slight decrease in *cpdA* expression in the *pilY1* mutant; however, when complemented, the activity of the reporter was not restored to wild-type levels (data not shown). There was also decreased expression of *cpdA* in the ∆*vfr* strain used as a control (57) (Fig. S3A). Altogether, these data suggest that Vfr only plays a significant role in regulating the phosphodiesterase *cpdA* and not the adenylate cyclases, *cyaA* and *cyaB*. Based on these data, PilY1 affects the expression of the major adenylate cyclase, *cyaB*. However, we cannot rule out that there is increased CyaB activity as well.

### AlgZ/R is responsible for increased cAMP levels in a *pilY1* mutant

A previous study demonstrated that two regulators, Vfr and the AlgZ/R two-component system, are increased in activity in a *pilY1* mutant (12). We investigated the role of Vfr and the AlgZ/R system in *cyaB* regulation to determine which regulator is involved in regulating *cyaB* expression. As shown in Fig. 3C, a double mutant containing a deletion in *pilY1* and encoding a phosphorylation-incompetent version of AlgR (∆*pilY1*RD54A) compared to the ∆*pilY1* strain suggests that phosphorylated AlgR is required for the increased *cyaB*. Luminescence from ∆*pilY1*RD54A was significantly decreased from *∆pilY1* (*P* < 0.0001, Table S3) but was not significantly different from PAO1. There was no difference between PAO1 and the *∆vfr* strain (Table S3). Even the wild-type strain had very little *cyaB-lux* reporter activity. These results suggest that phosphorylated AlgR, and not Vfr, was responsible for increased *cyaB* expression.

We constructed a second *cyaB* reporter using *lacZ* to further confirm the *lux* reporter data and further investigate if Vfr might play a role in *cyaB* expression. The ∆*pilY1* strain had a >3-fold increase (*P* < 0.001) in *lacZ* expression compared to the wild-type confirming the *lux* fusion data and the RNAseq data (Fig. 3D). Once again, introducing the allele encoding a phosphorylation-incompetent version of AlgR into the ∆*pilY1* strain (∆*pilY*1RD54A) abrogated the increased reporter activity (Fig. 3D). A ∆*vfr* strain did not differ from PAO1, the wild-type strain. When *vfr* was deleted in the ∆*pilY1* mutant (∆*pilY1*∆*vfr*), there was still almost a threefold increase in *lacZ* activity indicating that Vfr does not increase *cyaB* expression and is consistent with the *cyaB-lux* data (*P* < 0.001) (Fig. 3D). Additionally, the ∆*pilY1*∆*vfr* strain did not differ significantly from the ∆*pilY1* strain (Fig. 3D). These data indicate that AlgR, and not Vfr, is responsible for the increased *cyaB* expression in the ∆*pilY1* mutant.

To further support AlgZ/R regulation of *cyaB*, we constructed a ∆*algZ/R* strain that was complemented with the *algZ/R* operon under arabinose control. While no significant difference was found between PAO1 and ∆*algZ/R*, overexpression of the *algZ/R* operon resulted in increased *cyaB-lacZ* reporter activity (Fig. S4). These data further support the role of AlgZ/R in control of *cyaB* expression.

**Fig 4:**
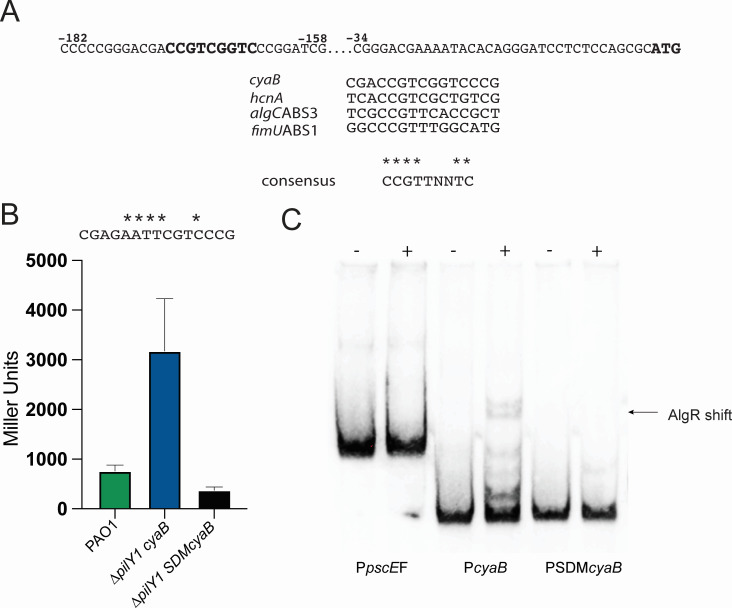
Site-directed mutagenesis of the AlgR-binding site of the *cyaB* promoter abolishes increased expression. (**A**) Sequence of the *cyaB* promoter region. The putative AlgR-binding site is indicated in bold. Below is an alignment of the putative AlgR-binding site compared to other AlgR binding sites. The consensus sequence is shown below. (**B**) Transcriptional fusion analysis of the *cyaB* promoter after site-directed mutagenesis. Sequence changes made to the putative AlgR-binding site are indicated above the sequence by asterisks. The *∆pilY1* SDMcyaB (far right) contains the mutagenized *cyaB-lacZ* fusion. (**C**) EMSA demonstrating the importance of the single AlgR-binding site in the *cyaB* promoter. PSDMcyaB is the mutated *cyaB* promoter (far right) used in the EMSA. A one-way ANOVA with Tukey’s post-test was used for statistical significance (***, *P* < 0.0001).

A putative AlgR-binding site was found in the *cyaB* upstream sequence (Fig. 4A). To determine if AlgR directly controlled *cyaB* expression by binding this sequence, purified AlgR (0.1 μM) was tested with the *cyaB* promoter using gel shift analysis (Fig. 3E and F). As a negative control, the *pscEF* genes were used. As a positive control, the *fimU* promoter (which also regulates *pilY1* expression) was also included. A shift comparable to the *fimU* positive control was evident (Fig. 3E). Competition experiments using the unlabeled *cyaB* promoter confirmed that the binding of AlgR to the *cyaB* promoter was specific (Fig. 3F). The gel shift studies indicated that AlgR directly binds the *cyaB* promoter.

The putative AlgR-binding site was located 170-162 bp upstream (Fig. 4A). The putative binding site only deviated at one nucleotide from the consensus sequence “CCGTTNNTC” (24) (Fig. 4A). We used site-directed mutagenesis to mutate the AlgR-binding site in the *cyaB-lacZ* transcriptional fusion (Fig. 4B) to determine if AlgR might bind to this site. Mutation would abolish AlgR activation if AlgR bound this site and provides an *in vivo* relevance of this promoter region. Once again, *cyaB* expression was more than threefold higher in activity in the ∆*pilY*1 mutant when using the wild-type *cyaB* promoter. However, the site-directed fusion had a greater than 4.5-fold reduction from the ∆*pilY*1 parent strain. Interestingly, mutation of the AlgR binding site in the fusion also reduced the reporter activity to approximately half of the wild-type strain PAO1 but was not significant (Fig. 4B).

Gel shift analysis was used to further define the nucleotides necessary for AlgR binding. We compared the native *cyaB* promoter to the site-directed mutagenized promoter. The native promoter bound AlgR as previously shown (Fig. 4C). However, the mutagenized promoter was unable to bind to AlgR (Fig. 4C). These data clearly demonstrate that AlgR directly regulates the *cyaB* promoter and indicate that the putative AlgR-binding site is legitimate. Therefore, the AlgZ/R system is important in the regulation of cAMP levels in *P. aeruginosa* by controlling *cyaB* expression. Overall, our data indicate that PilY1 is necessary to prevent AlgZ/R activation of *cyaB*.

### PilY1 functions in multiple strain types, including in mucoid strains

**Fig 5:**
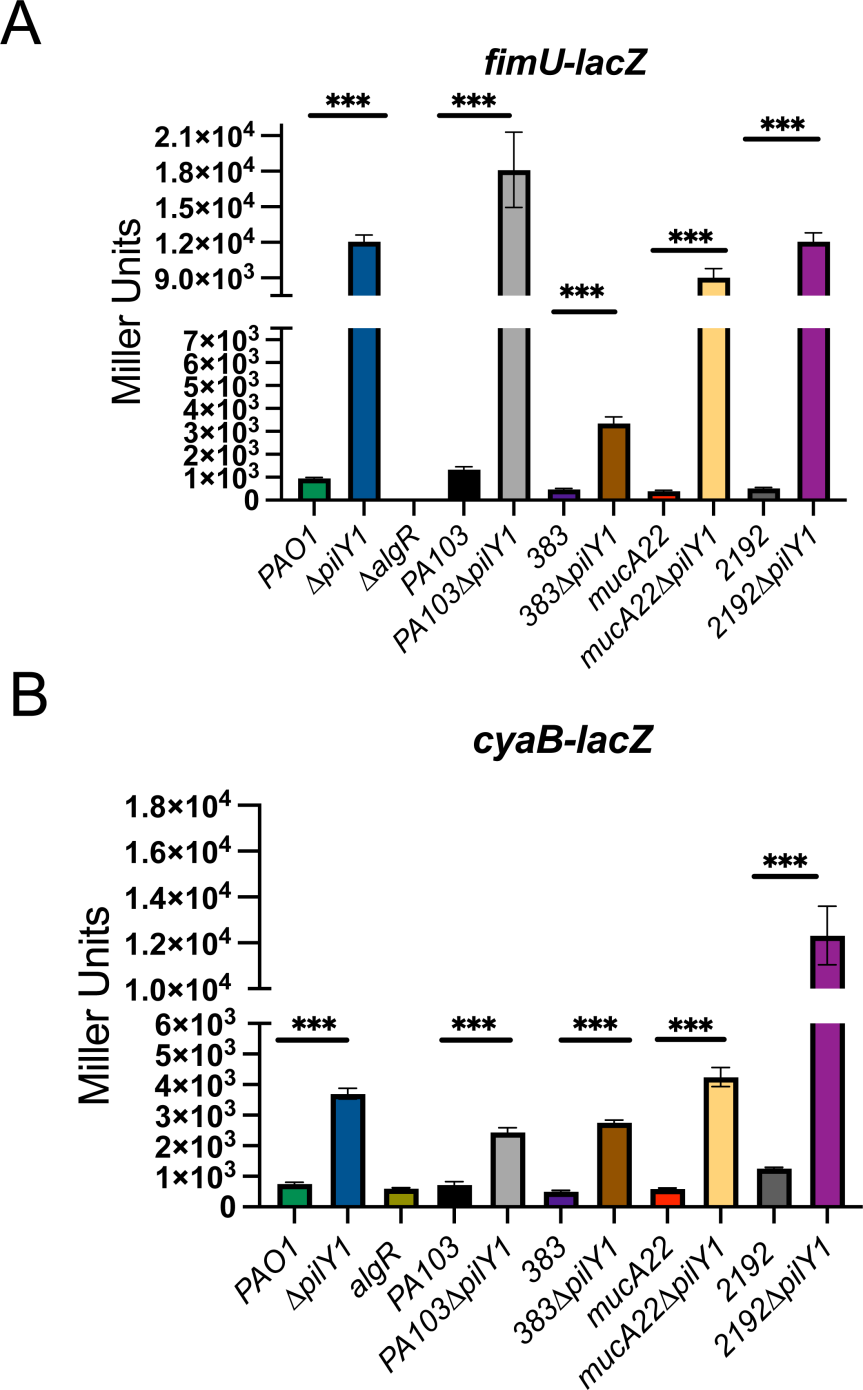
PilY1 functions similarly in both laboratory and clinical of *P. aeruginosa* strains. (**A**) Beta-galactosidase activity of a *fimU-lacZ* reporter to indicate that PilY1 is functional in five strains of *P. aeruginosa*. (**B**) Beta-galactosidase activity of a *cyaB-lacZ* reporter in multiple *P. aeruginosa* strains to demonstrate PilY1 functionality. Transcriptional fusions were performed in triplicate at least three times. *P* <****, 0.0001 based on Student’s *t*-test comparing the parent and the mutant strain.

Given the diversity of *P. aeruginosa* strains, we wanted to assess the functionality of PilY1 in other backgrounds. We used another strain PA103 (67) as this strain is representative of strains causing high rates of pneumonia and sepsis (68–72). We also used clinical strains, strain 383, and its mucoid derivative 2192 (73), and PDO300, the *mucA* mutant of PAO1. A *fimU-lacZ* transcriptional fusion was used to assess the functionality of PilY1 in these strains as previous studies have shown that *pilY1* mutation led to increased expression of its own operon (12, 29). A *∆algR* strain was used as a negative control as the AlgZ/R system positively regulates the *fimU* operon (21). As expected, there was a >20-fold increase in *fimU* expression in ∆*pilY1* vs PAO1 (Fig. 5A). Analysis of strain PA103 also resulted in increased *fimU* expression (*P* < 0.0001) when *pilY1* was deleted in this background (Fig. 5A). There was a sevenfold difference between the 383*∆pilY1* strain and the parental strain 383 for *fimU* expression (*P* < 0.0001) (Fig. 5A). Overall, these data suggest that PilY1 plays an important role in inhibiting the AlgZ/R system in numerous strains.

In cystic fibrosis patients, *P. aeruginosa* frequently loses motility and persists as a biofilm (74). Whether pili components, such as PilY1, are produced in mucoid strains has not been investigated. The rationale for these experiments was that PilY1 in a chronic *P. aeruginosa* isolate had significant phenotypic changes (32), suggesting that PilY1 can function independently of being incorporated into the pilus. The laboratory strain PDO300 (derived from PAO1) and a clinical isolate 2192 (75), which both contain mutations in *mucA*, were used. The *fimU* reporter had increased activity (>9-fold) in the *pilY1* mutants in both PDO300 and 2192 (Fig. 5A). These results suggested that PilY1 does function in *mucA* mutants found in chronic infections.

We also wanted to determine if AlgZ/R regulation of *cyaB* was also conserved in *P. aeruginosa* strains. The *cyaB-lacZ* transcriptional fusion was tested in various strains to monitor *cyaB* expression. There was increased activity of the fusion whenever a strain had been deleted for *pilY1* (Fig. 5B). Both PAO1*∆pilY1* and 383∆*pilY1* had increased *cyaB* reporter activity compared to their respective parent strains (Fig. 5B). We also saw an increase in *cyaB-lacZ* activity in the mucoid *∆pilY1* strains in both genetic backgrounds (Fig. 5B). When *pilY1* was deleted in PDO300 (PDO∆*pilY1*), there was a ~7-fold increase in *cyaB* reporter expression. In the case of 2192∆*pilY1,* there was an ~10-fold increase (Fig. 5B). This further confirms PilY1 is functional in *mucA* mutants and indicates that the signaling cascade is found in more than just the laboratory strain PAO1. These data also support that a major role of PilY1 is to repress the AlgZ/R system to prevent improper cAMP levels.

### PilY1 affects *algZ/R* expression

**Fig 6:**
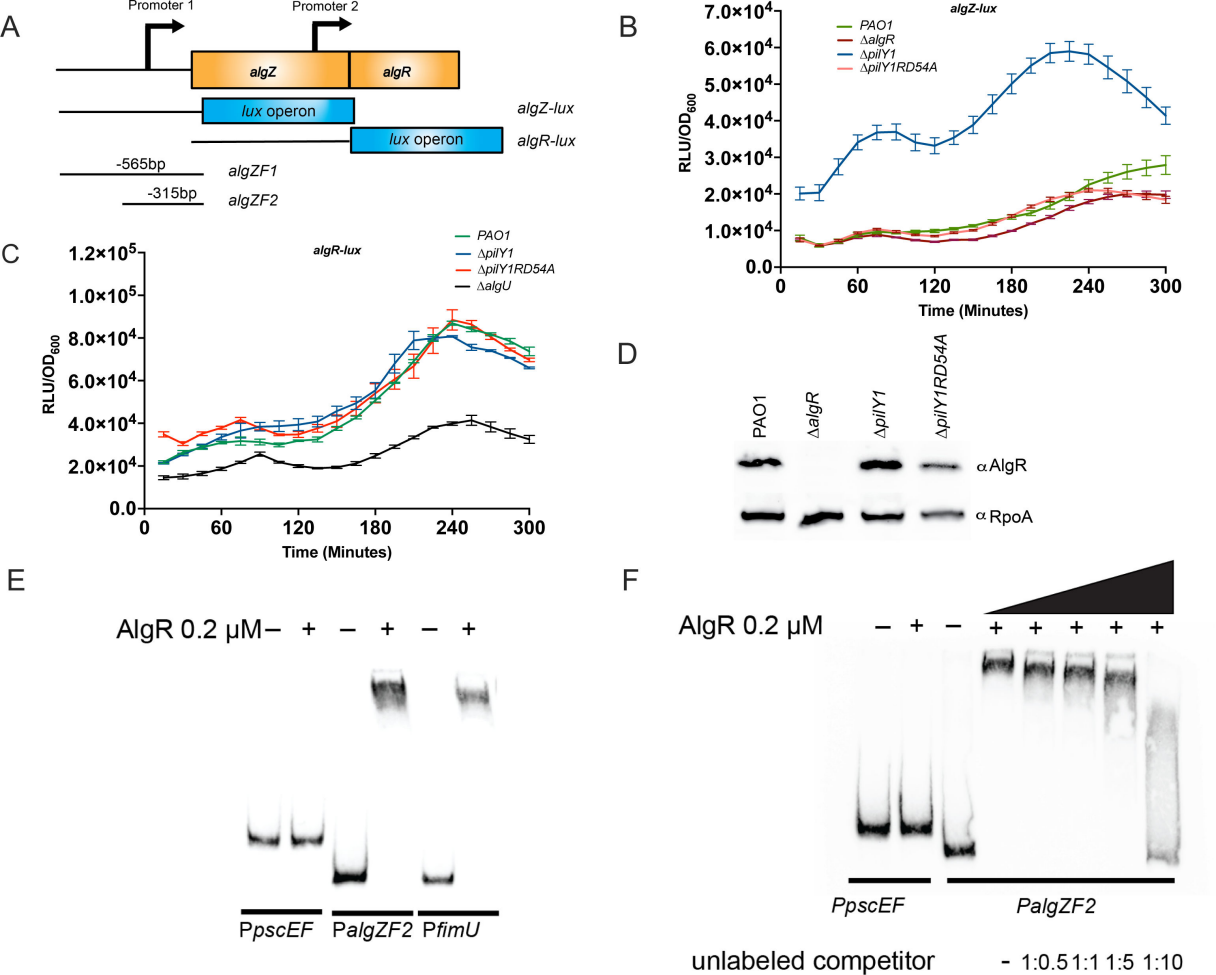
PilY1 prevents autoregulation of the *algZ/R* operon. (**A**) Schematic depicting the transcriptional fusions and the EMSA probes used. (**B**) An *algR-lux* transcriptional fusion tested in the *pilY1* mutant strains indicating no change in expression levels. (**C**) An *algZ-lux* transcriptional fusions tested in *pilY1* mutant strains showing increased *algZ* promoter activity in the ∆*pilY1* background. (**D**) Western blot analysis confirms increased AlgR in the ∆*pilY1* strain and decreased AlgR in the ∆*pilY1*RD54A strain. Ten micrograms of protein was loaded in each lane. A ∆*algR* strain was used as a negative control. RpoA was used as a loading control. (**E**) EMSA showing AlgR binding to the *algZ* promoter region. Purified AlgR bound both promoter probes algZF1 and algZF2. Shown is the EMSA using the shorter probe algZF2. (**F**) Competition EMSA showing AlgR specificity for *algZ* fragment 2. Transcriptional fusions were performed in triplicate at least three times. Western blotting was performed four times. A representative western is shown. EMSAs were performed at least three times. A representative EMSA is shown.

A previous study indicated that the AlgZ/R system was hyperactive in a ∆*pilY1* mutant, and this was responsible for attenuation in a *C. elegans* model (29). Additionally, our RNAseq data indicated increased *algZ* expression in the ∆*pilY1* strain. This suggested to us that the *algZ/R* operon might be subject to autoregulation, a common characteristic of two-component systems (76), but has not been directly tested for the *algZ/R* system. The *algZ/R* operon is under complex genetic regulation where promoters lie upstream of *algZ* and within the *algZ* coding region (77) (Fig. 6A). At least one of the promoters upstream of *algZ* (Promoter 1) is regulated by Vfr (20, 77). The promoters within the *algZ* coding region controlling only *algR* expression are activated by the alternative sigma factor AlgU/T (77). To begin to investigate autoregulation of the *algZ/R* promoters, we constructed *algZ* and *algR* transcriptional fusions to separate the two promoter regions (Fig. 6A) and assayed the activity in the minor pilin mutant ∆*pilY1*. Confirming the RNAseq data, the *pilY1* mutant had significantly increased activity of the *algZ* transcriptional fusion (*P* < 0.0001, Table S3), but there was no increased activity of the promoter located within the *algZ* coding region that controls only *algR* expression (Fig. 6B and C). If the minor pilin mutant has increased AlgZ/R activity, then this suggests that AlgR should exist in a phosphorylated state. When a double mutant consisting of the phosphodeficient allele of *algR* in the ∆*pilY1* background (∆*pilY*1RD54A) was analyzed with the *algZ* transcriptional fusion, the reported luminescence was not significantly different from the wild-type (Fig. 6B; Table S3). The transcriptional fusion analyses indicate that AlgR activates the expression of the *algZ*/*R* operon, but only from the most distal promoter upstream of *algZ*. Interestingly, Vfr was previously shown to regulate this same promoter (20, 78).

Western analysis was used to confirm increased AlgR levels in the *∆pilY1* mutant (Fig. 6D). AlgR was increased in the ∆*pilY1* strain compared to the wild-type strain PAO1 (Fig. 6D) indicating that *algR* is increased in *∆pilY1*. However, when the ∆*pilY*1RD54A strain was tested, AlgR levels were decreased compared to the ∆*pilY1* parental strain (Fig. 6D). A ∆*algR* strain was used as a negative control and RpoA was used as a loading control. These results suggested that increased expression of the *algZ/R* operon results in increased AlgR levels.

 To determine if AlgR activation of the *algZ* promoter was direct, we performed gel shift analyses using biotinylated probe fragments representing the promoter region upstream of *algZ*. Purified AlgR (0.2 mM) was able to shift both a fragment containing 565 bp upstream sequence and a shorter fragment consisting of 315 bp of upstream sequence (data not shown and Fig. 6E). We used the 315 bp fragment in competition experiments and inhibited AlgR binding using increasing concentrations of the unlabeled probes for the algZF2 probe (Fig. 6F). These results indicated that AlgR specifically binds to the *algZ* promoter.

### PilY1 affects PA4781 expression by preventing AlgR phosphorylation

PilY1 signals through the diguanylate cyclase SadC to increase c-di-GMP levels (19), and this explains why *∆pilY1* strains have decreased c-di-GMP. However, it is also possible that PilY1 affects c-di-GMP level by other means as well. The RNAseq results demonstrated increased *PA4781* transcripts in the ∆*pilY1* strain. *PA4781* was previously suggested to degrade c-di-GMP (79). We hypothesized that *PA4781* might contribute to the decreased c-di-GMP levels in the ∆*pilY1* mutant by degrading c-di-GMP.

**Fig 7:**
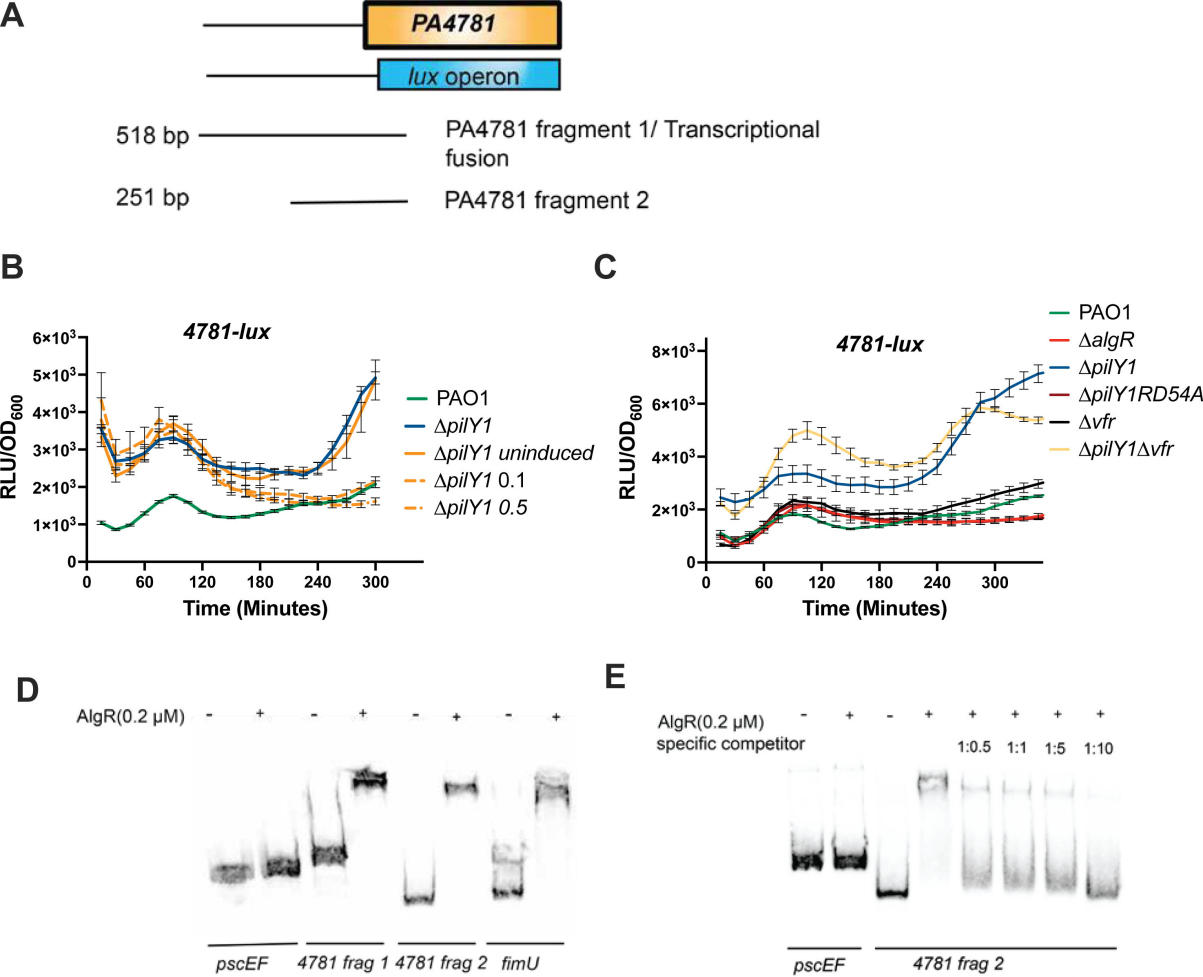
*PA4781* expression. (**A**) Schematic depicting the *PA4781* transcriptional fusion and fragments of the *PA4781* promoter region used for EMSA analysis. (**B**) Complementation of PilY1 reduces *PA4781* expression. A *PA4781* transcriptional fusion was tested in a complemented strain consisting of *pilY1* under an arabinose-inducible promoter. Percentages to the right (0.1 and 0.5) indicate the percentage of arabinose used. (**C**) Testing the *PA4781*-lux fusion to demonstrate AlgR-dependence and Vfr independence. (**D**) EMSA demonstrating that AlgR binds to the PA4781 promoter region. (**E**) Competitive EMSA showing specificity of AlgR binding to the *PA4781* promoter (4781 frag 2). Transcriptional fusions were performed in triplicate at least three times. EMSAs were performed at least three times. A representative EMSA is shown.

We first wanted to determine how PilY1 controls *PA4781*. We constructed a PA4781 transcriptional fusion (Fig. 7A and B). Confirming the RNAseq data, *PA4781* expression was significantly increased in a ∆*pilY1* (*P* < 0.0001, Table S3). Complementation of the ∆*pilY1* mutant with a single copy of *pilY1* and induction of 0.1% arabinose (or 0.5%) was able to fully restore levels to the wild-type after 120 min (Fig. 7B). Therefore, PilY1 affects the expression of *PA4781*.

We also analyzed the *PA4781-lux* fusion in strains to understand how *PA4781* is controlled in the context of PilY1. Because the AlgZ/R system and Vfr are important regulators in the ∆*pilY1* background (12), we investigated these regulators. When the allele of *algR* that encodes a phosphorylation-incompetent form of AlgR in the *pilY1* mutant was tested (∆*pilY1*RD54A), *PA4781* expression was significantly decreased from *∆pilY1* (Table S3), suggesting that phosphorylated AlgR is required for the activation of the *PA4781* promoter (Fig. 7C). A ∆*algR* mutant was also tested and had similar expression levels to the wild-type strain. We also tested this fusion in the ∆*vfr* and ∆*pilY1*∆*vfr* double mutant to determine if Vfr might also regulate *PA4781* expression. Mutation of *vfr* did not play a role in regulating *PA4781* compared to the wild-type (Fig. 7C). The *∆pilY1*∆*vfr* mutant had similar *PA4781-lux* activity as the *∆pilY1* mutant and was not statistically different suggesting that increased *PA4781* expression is not due to Vfr (Table S3). These data suggest that PilY1 prevents the AlgZ/R system from activating *PA4781* expression and that phosphorylated AlgR is required for transcriptional activation.

To determine if AlgR directly regulates *PA4781*, we performed gel shift analyses. Biotinylated PCR products were used to determine if AlgR directly bound to the PA4781 promoter region. A putative AlgR-binding site was located 425 bp upstream of *PA4781*. Two probes were made and incubated with 0.2 μM of purified AlgR (Fig. 7A). A probe containing 581 bp of upstream sequence bound purified AlgR that contained a potential AlgR-binding site. A shorter probe encompassing 251 of upstream sequence also bound AlgR even though no AlgR consensus sequence was found in this region (Fig. 7D). Competition experiments with the smaller upstream fragment using unlabeled probe were able to reduce AlgR binding indicating a specific interaction of AlgR with the shorter promoter fragment (Fig. 7E). From these data, we conclude that AlgR directly controls the expression of *PA4781*. Altogether, PilY1 decreases *PA4781* expression by preventing AlgR activation.

### PilY1 does not contribute to fitness in an acute pneumonia model

**Fig 8:**
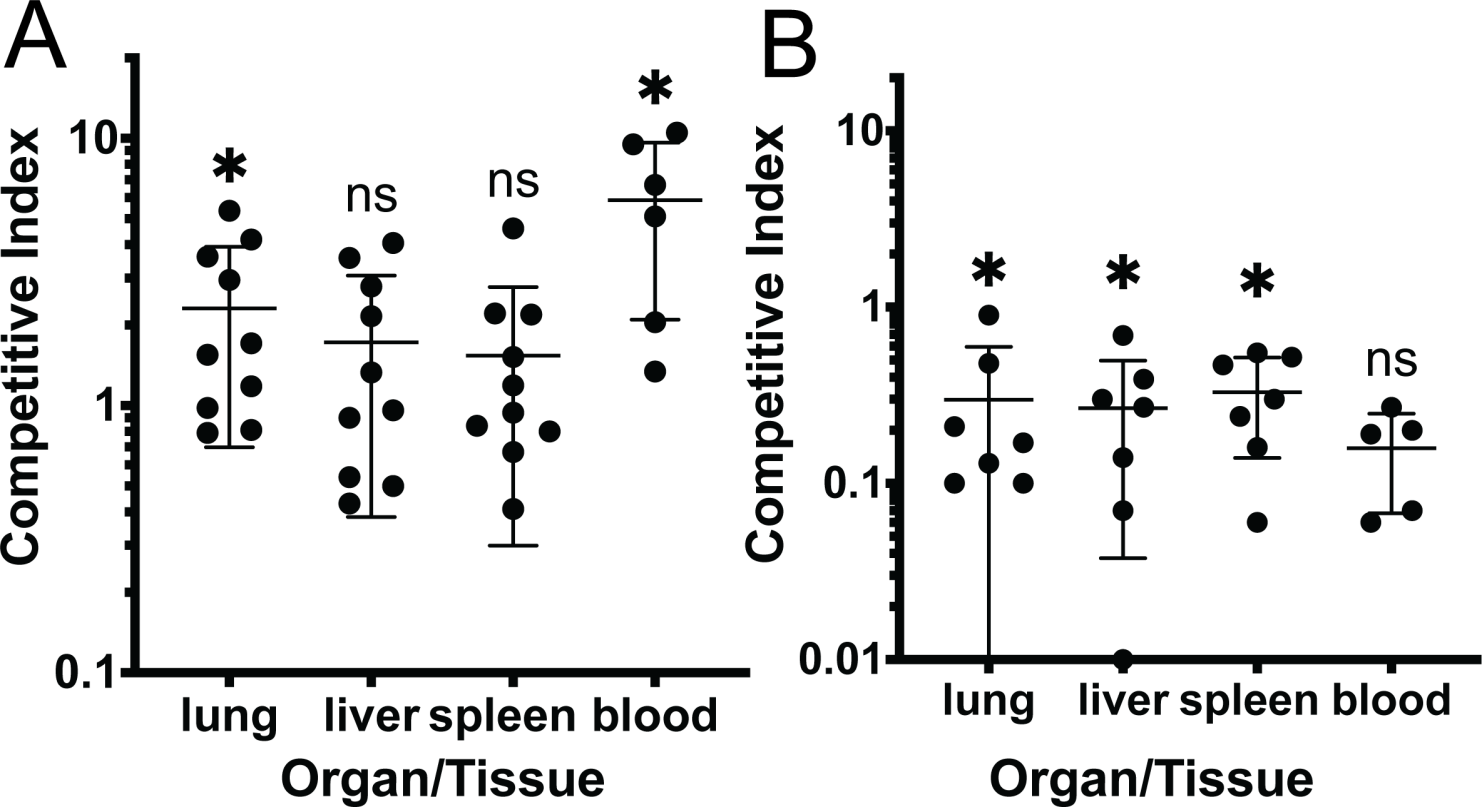
Assessment of PilY1 in *P. aeruginosa* virulence in an acute pneumonia model. Mice were infected for 16 h via oropharyngeal aspiration. Each point represents a competitive index. *n* = 10 for PAO1/∆*pilY1* and *n* = 7 for PAO1/∆*pilA*. (**A**) Competitive index of ∆*pilY1* vs PAO1 during infection. Four mice had no detectable colonies from the blood. (**B**) Competitive index of ∆*pilA* vs PAO1 during infection. Two mice had no detectable bacteria in blood (∆*pilA*/PAO1). One mouse liver was not used due to technical error. Statistical analysis was using the Wilcoxon signed rank test. *, *P* < 0.05.

Several studies have indicated that *pilY1* mutants are attenuated (10, 29, 32, 80). However, only one of these studies used a mouse model. The strain used was an uncharacterized clinical isolate, and the bacteria were encased in alginate beads to model a chronic infection (32). We tested the ∆*pilY1* strain (derived from the PAO1 background) in a competition experiment with the wild-type strain to determine the fitness of this strain in an acute pneumonia. Both PAO1 and the ∆*pilY1* strains were labeled using either a gentamicin resistance cassette (PAO1) or a tetracycline resistance cassette (∆*pilY1*). The PAO1 and ∆*pilY1* strains were mixed in a 1:1 ratio at a dose of 1 × 10*^7^* CFU/mouse (a lethal dose at 24 h). We expected the *∆pilY1* mutant to be less competitive to the wild-type based on previous studies. After 16 h of infection, the lungs, spleen, and liver were homogenized and plated on differential media to distinguish between the wild-type and mutant to calculate the competitive index (Fig. 8). Blood was also analyzed; however, there were no bacteria detected in the blood from 4 mice. Results showed that the ∆*pilY*1 mutant outcompeted the wild-type as indicated by CI values greater than 1.0 in the lung (CI = 1.63) and blood (CI = 5.9) (Fig. 8A). However, in the liver and spleen, the CI was close to 1 indicating that the ∆*pilY*1 mutant was able to compete as well as PAO1 in these tissues (CI = 1.15 and 1.1, respectively) (Fig. 8A). Overall, these results suggest that the ∆*pilY*1 mutant is more competitive in certain tissues and at least as competitive as the wild-type strain using the acute pneumonia model.

 The type IV pili mediate close interaction of bacteria with host cells in *in vitro* studies, but few studies have tested if type IV pili are important in pathogenesis *in vivo*. We tested a ∆*pilA* mutant in a competition experiment as described for ∆*pilY1*. In contrast to the ∆*pilY1* data, a ∆*pilA* mutant was attenuated in the lung, liver, and spleen (Fig. 8B). The CI of ∆*pilA* was not significantly different in the blood, but this was likely because we were only able to enumerate CFUs from five mice. Altogether, these data indicate that a ∆*pilA* mutant is attenuated in the acute pneumonia model.

## DISCUSSION

** **PilY1 is an adhesive component of the T4P and is important in mechanosensing (7, 10, 12, 29, 32, 81). However, there are still questions regarding the role of PilY1 in pathogenesis and the outcome of PilY1 signaling. Previous studies have established that PilY1 prevents activity of the AlgZ/R two-component system (12, 29). Therefore, a ∆*pilY1* strain provides an opportunity to determine the role of phosphorylated AlgR in *P. aeruginosa*. Here, we identify new AlgZ/R-controlled genes using a ∆*pilY1* strain. We found that the expression of the major adenylate cyclase, *cyaB,* and an unannotated putative cyclic di-GMP phosphodiesterase, PA4781, are increased in a ∆*pilY1* mutant. We demonstrate that both *cyaB* and *PA4781* are directly controlled by the AlgZ/R two-component system identifying new members of the AlgZ/R regulon. This signaling cascade was conserved in multiple *P. aeruginosa* strains. Additionally, we demonstrate that PilY1 plays a nuanced role in virulence in the acute pneumonia model. Overall, our study determined new members of the AlgZ/R regulon and demonstrated genes that are dysregulated in a ∆*pilY1* strain.

Using transcriptional fusion analyses, gel shift studies, and site-directed mutagenesis, we firmly establish that the AlgZ/R system can activate *cyaB* expression. Control of cAMP levels is important to allow bacteria to move from a planktonic lifestyle to a biofilm lifestyle (37, 38). Other pili components control the activity of CyaB after expression (11, 12, 56), but this would occur after PilY1 has bound to a surface. It may be that there is expression of *cyaB* in the planktonic phase, but the CyaB enzymatic function is not active until the T4P extend and contract after binding a surface. This would help explain the surface-activated virulence seen previously (10).

The AlgZ/R system was also found to be subject to autoregulation. Previous studies have alluded to this autoregulation (82, 83), but no direct test of this has been done to date. The results presented here demonstrate direct regulation of the *algZ/R* operon by AlgR. Additionally, these data demonstrate that phosphorylated AlgR binds to the promoter upstream of *algZ*. Therefore, PilY1 control of the AlgZ/R system results in decreased expression of this system as well as decreased activity. Further work is necessary to determine how PilY1 prevents AlgR phosphorylation.

Our study also determined that the AlgZ/R system controls the expression of the putative phosphodiesterase, *PA4781*. The role of PA4781 in controlling c-di-GMP is still unclear (79, 84). While we cannot ascribe a role for PA4781, we have aided in understanding its transcriptional regulation. Understanding how PA4781 is involved in secondary messenger degradation may be facilitated by using a *pilY1* mutant strain where its expression, and perhaps activity, is increased.

Our use of the *∆pilY1* strain provides a more physiological context to decipher AlgR function than other studies. None of the genes in our study were found in previous investigations studying AlgR using microarray or ChiP-seq analysis (82, 85). A possible explanation for why our study detected these changes in *cyaB*, *algZ*, and *PA4781* is our use of the ∆*pilY1* strain that allows increased phosphorylated AlgR (29). Previous studies may not have had phosphorylated AlgR whether using an *algR* overexpression strain (82) or purified AlgR (85). The stability of the acyl-phosphate bond in response regulators can be unstable and can have half-lives of seconds to hours (86, 87). The lack of phosphorylated AlgR in the previous studies could explain why these genes were not identified in those studies.

The role of PilY1 in virulence is still unclear. Previous studies have indicated that PilY1 is important in virulence (10, 29, 32). Only one of the previous studies used a mouse model of infection. This study used a chronic isolate and chronic infection model using alginate beads and only examined the lungs. However, the authors also found an increase in CFUs of the *∆pilY1* strain compared to the wild-type in a competition experiment (32) similar to our study. The competitive index (CI) is a sensitive measure of comparing virulence between mutant and wild-type strains (88). We found that the *∆pilY1* strain was at least as fit in the liver and spleen and more fit than the wild-type in the lungs and blood. While we did not evaluate the immunopathology during these infections, the fact that the host is incapable of eliminating the *∆pilY1* strain complicates assigning the *∆pilY1* strain as attenuated. It may be that the timepoint used in the acute pneumonia model of 16 h was not sufficient to see a difference between the two strains. For instance, if the *∆pilY1* strain caused more neutrophil infiltration, this could result in more pathology early on, but later timepoints might find that the mutant strain is eliminated at greater numbers than the wild-type.

A previous study indicated that a *pilA* mutant was attenuated in a survival study (9). Our work confirms this result using a slightly different model and a different strain. Using oropharyngeal aspiration instead of intranasal inoculation to introduce bacteria ensures more bacteria in the lungs (89, 90). This makes our model relevant to the study of ventilator-associated pneumonia. A recent transcriptomic analysis of a ∆*pilA* strain suggested that the main reason for attenuation is due to cAMP production (91). It is possible that the dysregulated cAMP levels in the ∆*pilY1* are responsible for the increased virulence. Given the importance of careful regulation of second messengers, inappropriate levels at a given time may attenuate *P. aeruginosa* virulence. Additionally, our study has demonstrated that while some pili components are required for virulence, such as PilA, others, such as PilY1, can be unnecessary after the colonization phase.

**Fig 9:**
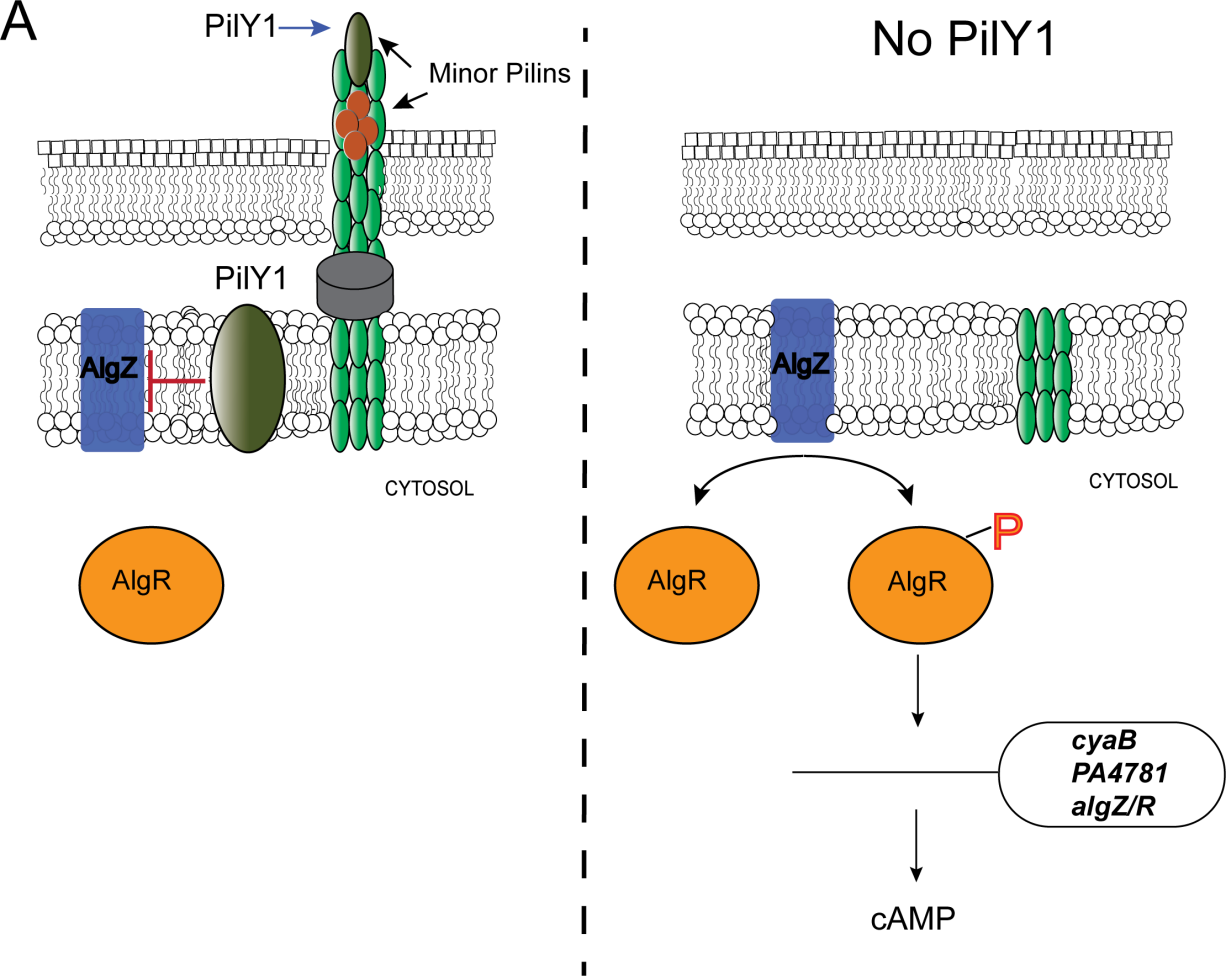
PilY1 exerts its effects by turning off the AlgZ/R system. (**A**) PilY1 turns “off” AlgZ/R to prevent increased *cyaB*, *PA4781*, and *algZ/R* expression. PilY1, through unknown mechanisms, prevents AlgZ phosphorylation of AlgR. Mutation of *pilY1* results in increased AlgR phosphorylation that causes aberrant gene expression. This study uncovered new AlgZ/R targets and suggests that PilY1 signaling requires turning off expression of *cyaB*, *PA4781*, and the *algZ/R* operon to allow *P. aeruginosa* to modify its lifestyle upon surface contact.

Based on our data and the work of others, we propose that the major function of PilY1 is to regulate the activity of the AlgZ/R system (Fig. 9). In the case of *pilY1* expression, phosphorylated AlgR and Vfr activate the *fimU* operon (which includes *pilY1*) (20, 21). The work presented here suggests that the AlgZ/R system can also activate *cyaB* expression allowing cAMP production that can then activate Vfr. Phosphorylated AlgR and Vfr can then activate the *fimU* operon (including *pilY1*). After the expression of the *fimU* operon, PilY1 turns off the AlgZ/R system. This would then allow Vfr to activate other targets such as the T3SS. Given that AlgR can inhibit T3SS expression (92) and that the T3SS is activated via surface contact (93, 94), PilY1 deactivation of AlgZ/R is required for Vfr to activate the T3SS. Could phosphorylated AlgR be used to prevent Vfr from activating the T3SS expression? This could explain the reduced virulence of *algR* overexpressing strains previously described (95). In addition, does PilY1 continue to turn off the AlgZ/R system when PilY1 is engaged with its cognate ligand on a host cell? Further studies using *pilY1* variants instead of deletion mutants are required to answer these questions.

PilY1 has garnered attention for its role in mechanosensing and virulence. In this report, we have identified an important role of PilY1 in controlling cAMP as well as c-di-GMP. This aids our understanding of how the T4P can engage in signaling but also suggests that PilY1 function is important in other locations as well. We also have aided in the understanding of how different regulatory pathways intersect. Vfr and AlgZ/R can work together to activate expression of the *fimU* operon. Once *pilY1* is expressed, PilY1 turns off the AlgZ/R system and other pili components can activate CyaB allowing Vfr to activate expression of virulence genes. Further work is necessary to unravel the role of PilY1 in *P. aeruginosa* pathogenesis. Understanding signal transduction networks can identify new therapeutic targets and allow new ways to treat *P. aeruginosa* and other antibiotic-resistant bacteria.

## SUPPLEMENTAL MATERIAL

Supplemental Material jb.00200-25-s0001.docx Tables S1 to S3; Fig. S1 to S4.

Dataset S1 jb.00200-25-s0002.xlsx Upregulated and downregulated genes.

ASM does not own the copyrights to Supplemental Material that may be linked to, or accessed through, an article. The authors have granted ASM a non-exclusive, world-wide license to publish the Supplemental Material files. Please contact the corresponding author directly for reuse.
