# Supplementary material for: Defining the role of Pseudomonas aeruginosa PilY1 in signaling and virulence
Source: J Bacteriol. 2026 Feb 9;208(3):e00200-25. doi: 10.1128/jb.00200-25 (PMC13001225; doi:10.1128/jb.00200-25)
Supplement: Supplemental Material — Tables S1 to S3; Fig. S1 to S4. [file jb.00200-25-s0001.docx]

**Supplementary materials for:**

**Defining the role of *Pseudomonas aeruginosa* PilY1 in signaling and virulence.**

Christopher L. Pritchett^a#^, F.H. Damron^bc^, Mariette Barbier^bc^

^a^Department of Biomedical Health Sciences, College of Public Health, East Tennessee State University, Johnson City, TN

^b^ Department of Microbiology, Immunology, and Cell Biology, West Virginia University, Morgantown, WV

^c^ Vaccine Development Center at West Virginia University Health Sciences Center, Morgantown, WV

^#^corresponding author: Christopher L. Pritchett ([pritchettc@etsu.edu](mailto:pritchettc@etsu.edu))

**Table of Contents**

**Table S1**. Strains and Plasmids……………………………………………………………………….2-4

**Table S2**. Primers……………………………………………………………………….......................5-6

**Table S3**. Statistics for AUC comparisons…………………………………………………………….7

**Figure S1**. Complementation of ∆*pilY1*………………………………………………………………..8

**Figure S2**. Complementation of ∆*pilY1 cdrA-lux* with pPilY1…….……………..…………………..9

**Figure S3**. Transcriptional analysis of cAMP components to plain increased cAMP in ∆*pilY1*….10

**Figure S4**. Overexpression of *algZ/R* increases *cyaB-lacZ* reporter activity………………………11

**References**………………………………………………………………………………………………..12

Table S1. Strains and plasmids

| Strain or Plasmid | Genotype or Relevant properties | Reference |
| --- | --- | --- |
| *E. coli* strains |  |  |
| NEB5α | *fhuA2 Δ(argF-*lacZ*) U169phoA, glnV44 Φ80Δ(*lacZ*)*M*15 gyrA96* | New England Biolabs |
| SM10 | *Thi thr leu tonA lacY supE recA*::RP4-2-Tc::Mu Km^R^ | (1) |
| pRK2013 | Helper strain | (2) |
| pEX18Tc | Allelic exchange vector | (3) |
| pEX18Gm | Allelic exchange vector | (3) |
| ∆*pilY1* pEX18Tc | Allelic exchange for making *pilY1* mutant | This study |
| miniCTXlacZ | Transcriptional fusion vector |  |
| *algZ-lux* | Transcriptional fusion | This study |
| *algR-lux* | Transcriptional fusion | This study |
| *fimU-lacZ* | Transcriptional fusion | This study |
| *cyaB-lacZ* | Transcriptional fusion | This study |
| mini-CTX-*lux* | Transcriptional fusion vector |  |
| *lacP1-lux* | Transcriptional fusion | This study |
| *cdrA-lux* | Transcriptional fusion | This study |
| *cyaB-lux* | Transcriptional fusion | This study |
| PA4781-*lux* | Transcriptional fusion | This study |
| pTJ1 | Integrating vector used for overexpression | This study |
| *algR* pTJ1 (pAlgR) | *algR* complementation/overexpression | This study |
| *pilY1 pTJ1* | *pilY1* complementation/overexpression | This study |
| *P. aeruginosa*  strains |  | This study |
| PAO1 | Wild type | (4) |
| *∆alg*R | *algR* mutant | (5) |
| *mucA22* | *mucA22* mutation | (6) |
| *mucA22 ∆algR* | *mucA22* strain, ∆*algR* | (6) |
| PAO1 | PAO1 with epitope tagged AlgR | This study |
| D54A | *algR* phospho-deficient strain | This study |
| ∆*pilW* | *pilW* mutant | This study |
| ∆*pilWalgR*D54A | *pilW* mutant and *algR* phospho-deficient strain | This study |
| ∆*pilW*∆*algR* | *pilW* mutant and *algR* mutant | This study |
| ∆*pilA* | *pilA* mutant | This study |
| ∆*pilY1* | *pilY1* mutant | This study |
| ∆*pilY1*RD54A | *pilY1* mutant and *algR* phospho-deficient strain | This study |
| ∆*pilJ* | *pilJ* mutant | This study |
| *∆4781* | *PA4781* mutant | This study |
| ∆*pilY1*∆*4781* | *pilY1* mutant and *PA4781* mutant | This study |
| 383 | Clinical isolate | Gerald Pier; (7) |
| *383∆pilY1* | Clinical isolate with *pilY1* mutation | This study |
| *2192* | Clinical isolate | Gerald Pier; (7) |
| 2192∆*pilY1* | Clinical isolate with *pilY1* mutation | This study |
| ∆*gacA* | *gacA* mutant | (8) |
| ∆*cpdA* | *cpdA* mutant | This study |
| ∆*vfr* | *vfr* mutant | This study |
| *∆pilY1∆vfr* | *pilY1, vfr* doublemutant | This study |
| PAO1SDM | Mutated *cyaB* promoter | This study |
| ∆*pilY1*SDM | *pilY1* mutant with mutated *cyaB* promoter | This study |
| ∆*lasR∆rhlR* | *lasR, rhlR* double mutant | (9) |
| *∆algU* | *algU* mutant | (8) |
| PAO1 *cdrA-lux* | Transcriptional fusion | This study |
| *∆pilY1 cdrA-lux* | Transcriptional fusion | This study |
| *∆pilA cdrA-lux* | Transcriptional fusion | This study |
| *∆pilJ cdrA-lux* | Transcriptional fusion | This study |
| *∆gacA cdrA-lux* | Transcriptional fusion | This study |
| PAO1 *lacP1-lux* | Transcriptional fusion | This study |
| *∆pilY1 lacP1-lux* | Transcriptional fusion | This study |
| *∆pilA lacP1-lux* | Transcriptional fusion | This study |
| *∆pilJ lacP1-lux* | Transcriptional fusion | This study |
| *∆vfr lacP1-lux* | Transcriptional fusion | This study |
| *∆cpdA lacP1-lux* | Transcriptional fusion | This study |
| *∆pilIY1 pPilY1 cdrA-lux* | Transcriptional fusion | This study |
| *∆pilIY1 pPilY1 lacP1-lux* | Transcriptional fusion | This study |
| PAO1 PA4781*-lux* | Transcriptional fusion | This study |
| *∆pilY1 PA4781-lux* | Transcriptional fusion | This study |
| *∆pilIY1 pPilY1 PA4781-lux* | Transcriptional fusion | This study |
| *∆algR PA4781-lux* | Transcriptional fusion | This study |
| *∆pilY1RD54A PA4781-lux* | Transcriptional fusion | This study |
| *∆vfr PA4781-lux* | Transcriptional fusion | This study |
| *∆pilY1∆vfr PA4781-lux* | Transcriptional fusion | This study |
| *∆PA4781 lacP1-lux* | Transcriptional fusion | This study |
| *∆pilY1∆PA4781 lacP1-lux* | Transcriptional fusion | This study |
| *∆PA4781 cdrA-lux* | Transcriptional fusion | This study |
| *∆pilY1∆PA4781 cdrA-lux* | Transcriptional fusion | This study |
| *PAO1 algZ-lux* | Transcriptional fusion | This study |
| *∆algR algZ-lux* | Transcriptional fusion | This study |
| *∆pilY1 algZ-lux* | Transcriptional fusion | This study |
| *∆pilY1RD54A algZ-lux* | Transcriptional fusion | This study |
| *PAO1 algR-lux* | Transcriptional fusion | This study |
| *∆algR algR-lux* | Transcriptional fusion | This study |
| *∆pilY1 algR-lux* | Transcriptional fusion | This study |
| *∆pilY1RD54A algR-lux* | Transcriptional fusion | This study |
| *PAO1 cyaB-lux* | Transcriptional fusion | This study |
| *∆algR cyaB-lux* | Transcriptional fusion | This study |
| *∆pilY1 cyaB-lux* | Transcriptional fusion | This study |
| *∆pilY1RD54A cyaB-lux* | Transcriptional fusion | This study |
| *∆pilIY1 pPilY1 cyaB-lux* | Transcriptional fusion | This study |
| *∆pilY1RD54A cyaB-lux* | Transcriptional fusion | This study |
| *∆vfr cyaB-lux* | Transcriptional fusion | This study |
| *PAO1 cyaB-lacZ* | Transcriptional fusion | This study |
| *∆algR cyaB-lacZ* | Transcriptional fusion | This study |
| *∆pilY1 cyaB-lacZ* | Transcriptional fusion | This study |
| *∆pilY1RD54A cyaB-lacZ* | Transcriptional fusion | This study |
| *∆pilIY1 pPilY1 cyaB-lacZ* | Transcriptional fusion | This study |
| *∆pilY1RD54A cyaB-lacZ* | Transcriptional fusion | This study |
| *∆vfr cyaB-lacZ* | Transcriptional fusion | This study |
| *∆pilY1∆vfr cyaB-lacZ* | Transcriptional fusion | This study |
| *PAO1SDM cyaB-lux* | Transcriptional fusion | This study |
| *∆pilY1SDM cyaB-lux* | Transcriptional fusion | This study |
| *PAO1SDM cdrA-lux* | Transcriptional fusion | This study |
| *∆pilY1SDM cdrA-lux* | Transcriptional fusion | This study |
| *PAO1 fimU-lacZ* | Transcriptional fusion | This study |
| *383 fimU-lacZ* | Transcriptional fusion | This study |
| *∆pilY1 fimU-lacZ* | Transcriptional fusion | This study |
| *383∆pilY1 fimU-lacZ* | Transcriptional fusion | This study |
| *2192 fimU-lacZ* | Transcriptional fusion | This study |
| *2192∆pilY1 fimU-lacZ* | Transcriptional fusion | This study |
| *mucA22 lacP1-lux* | Transcriptional fusion | This study |
| *mucA22∆pilY1 lacP1-lux* | Transcriptional fusion | This study |
| *2192 lacP1-lux* | Transcriptional fusion | This study |
| *2192∆pilY1 lacP1-lux* | Transcriptional fusion | This study |
| *mucA22 cyaB-lacZ* | Transcriptional fusion | This study |
| *mucA22∆pilY1 cyaB-lacZ* | Transcriptional fusion | This study |
| *2192 cyaB-lacZ* | Transcriptional fusion | This study |
| *2192∆pilY1 cyaB-lacZ* | Transcriptional fusion | This study |
| *383 cyaB-lacZ* | Transcriptional fusion | This study |
| *383∆pilY1 cyaB-lacZ* | Transcriptional fusion | This study |
| *mucA22 cdrA-lux* | Transcriptional fusion | This study |
| *mucA22∆pilY1 cdrA-lux* | Transcriptional fusion | This study |
| *2192 cdrA-lux* | Transcriptional fusion | This study |
| *2192∆pilY1 cdrA-lux* | Transcriptional fusion | This study |
| *PA103* |  | (10) |
| *PA103∆pilY1* |  | This study |
| *PA103 fimU-lacZ* | Transcriptional fusion | This study |
| *PA103∆pilY1 fimU-lacZ* | Transcriptional fusion | This study |
| *PA103 cyaB-lacZ* | Transcriptional fusion | This study |
| *PA103∆pilY1 cyaB-lacZ* | Transcriptional fusion | This study |

Table S2. Primers used in this study

| Primer Name | Sequence | Use |
| --- | --- | --- |
| algRTJ1R | GCGCAAGCTTTCAGAGCTGATGCATCAGACGC | Overexpression |
| algRintF | GCAACTGGACTGGCAGGTGC | Mutant |
| algRintR | CGCGACTGGTCATCGGCAG | Mutant |
| algRBamHIR | GCGCGGATCCGTCAGAGCTGATGCATCAGACG | Mutant |
| algZHindIIIF | GCGCAAGCTTCTCTCGCTGCAACAAGAAACGG | Mutant |
| algZSDMcheckF | CAGCTGGGCGGAGAACTGAC | Mutant |
| algZHSDMF | GAATTCCTGTTCAACAGCCTGAACAG | Mutant |
| algZHSDMR | CGGGCGAATCCGCGCCTGCA | Mutant |
| lacZRforTF | GATGTGCTGCAAGGCGATTAAG | SEQ |
| pHERDSF | ATCGCAACTCTCTACTGTTTCTC | SEQ |
| cdrATFF | GCGCAAGCTT GCAGCTCGTCGAAGGCG | Transcriptional Fusion |
| cdrATFR | GCGCGGATCC TCGAAGAGGTGCGCTTGCC | Transcriptional Fusion |
| EclacP1F | GCGCGAATTC GCCCAATACGCAAACCGC | Transcriptional Fusion |
| EclacP1R | GCGCGGATCC TCAGGCGAAAGGGGGATGTGCTG | Transcriptional Fusion |
| algZSOER | GACATTCATAAGCTCAGGCTTCCCATCGACAGAGTTTCCGCAAGG | Mutant |
| vfrEcoRIF | GCGCGAATTCGACCACATCGAAGTTGGTGCAG | Mutant |
| vfrBamHIR | GCGCGGATCCGTAATAGCTACCATGCCCGAGTC | Mutant |
| pilinpTJ1F | GCGC GAA TTC GTC ATA TCG TTC CAA CTC GAC C | Complementation |
| pilinpTJ1R | GCGC TCT AGA TCA GCG CCA GCA GTC GTT GAC | Complementation |
| algRBamHIR | GCGCGGATCCGTCAGAGCTGATGCATCAGACG | Mutant |
| algZHindIIIF | GCGCAAGCTTCTCTCGCTGCAACAAGAAACGG | Mutant |
| algZHSDMF | GAATTCCTGTTCAACAGCCTGAACAG | Mutant |
| algZHSDMR | CGGGCGAATCCGCGCCTGCA | Mutant |
| pilY1F | GCGCGAGCTCCAGGAGATCGTCAGCGGCCTG | Mutant |
| pilY1R | GCGCGAATTCCAGCGCCAGCAGTCGTTGAC | Mutant |
| pilY1SOEF | CAACTGATCGAGCCTCGCATGTGATATGAAAGTGCTGCCTATGC | Mutant |
| pilY1SOER | GCATAGGCAGCACTTTCATATCACATGCGAGGCTCGATCAGTTG | Mutant |
| pilY1intF | CACGCTCTACAGCGTGCAGTG | SEQ |
| pilY1intR | GAAGACCACGCCAGCGCCT | SEQ |
| vfrF | GCGCAAGCTTGTTGTTGAACCCGTGGAGCTTG | Mutant |
| vfrR | GCGCTCTAGAGATCTGGCGACCAGCCTGCA | Mutant |
| vfrSOEF | CTTTCGGGACTCGGGCATGGAATTCTGAACAGCACCCATGAAAAACC | Mutant |
| vfrSOER | CCTTTTTCATGGGTGCTGTTCAGAATTCCATGCCCGAGTCCCGAAAG | Mutant |
| vfrintF | GTCCGTCTGTTAAGCTGGACG | SEQ |
| vfrintR | CTTCCAGGAGCGTGGTGCTG | SEQ |
| algRTJ1R | GCGCAAGCTTTCAGAGCTGATGCATCAGACGC | Complementation |
| algRTJ1F2 | GCGCCCATGGGAATGAATGTCCTGATTGTCGATGAC | Complementation |
| pilAR | GCGCGAATTCCCGCACCGCCCTGGCGG | Mutant |
| pilASOEF | CAACGGAGAGATTCATGAAAGCGATAACTAAGGTGATCGAAGG | Mutant |
| pilASOER | CCTTCGATCACCTTAGTTATCGCTTTCATGAATCTCTCCGTTG | Mutant |
| pilAintF2 | GACAGGCCGCTCAGTTGGATG | SEQ |
| pilAintR2 | GTCGACCTGTCCATGCGCCTC | SEQ |
| algRHindIIIF | GCGCAAGCTTCGATGCCTATCCGATTCAAGC | Mutant |
| algRintR | CGCGACTGGTCATCGGCAG | SEQ |
| pilJFBamHIF | GCGCGGATCCGAAGAAAATCAACGCAGGCAATC | Mutant |
| pilJXbaIR | GCGCTCTAGA GAGCGACGTAGTCGTGCCGG | Mutant |
| pilJSOEF | AATATGAAGAAAATCAACGCAGGCGTGGAGCAGGCCTGAGCATA | Mutant |
| pilJSOER | TATGCTCAGGCCTGCTCCACGCCTGCGTTGATTTTCTTCATATT | Mutant |
| pilJintF | CATGGCGTCTTCCATCGAGAG | SEQ |
| pilJintR | GTTCTCCAACAGGACCTGCCA | SEQ |
| algZSDMcheckF | CAGCTGGGCGGAGAACTGAC | SEQ |
| algZSDMcheckR |  | SEQ |
| algZSOEF | CCTTGCGGAAACTCTGTCGATGGGAAGCCTGAGCTTATGAATGTC | Mutant |
| algZSOER | GACATTCATAAGCTCAGGCTTCCCATCGACAGAGTTTCCGCAAGG | Mutant |
| pilY1HAF | TACCCATACGATGTTCCAGATTACGCTTGATATGAAAGTGCTGCCTATGCT | HA Mutant |
| pilY1HAR | TCAAGCGTAATCTGGAACATCGTATGGGTAGTTCTTTCCTTCGATGGGGCGC | HA Mutant |
| cpdATFFHindIII | GCGCAAGCTT CTACACCACCACCCTTCAGGT | Transcriptional Fusion |
| cpdATFRBamHI | GCGCGGATCC GCACCAGCAGTACGGAGGCG | Transcriptional Fusion |
| cyaATFFHindIII | GCGCAAGCTT GTACCAGAAGATCCTCGACGG | Transcriptional Fusion |
| cyaATFRBamHI | GCGCGGATCC ATCGTCGAGATCGAGGCTGAG | Transcriptional Fusion |
| cpdASOEF | CTGCTGGTGCAGCTCGAATTCGAAGTGGACTACGACACCG | Mutant |
| cpdASOER | CGGTGTCGTAGTCCACTTCCAATTCGAGCTGCACCAGCAG | Mutant |
| cyaBHindIIF | GCGCAAGCTT GATGATCCGCTGGTAGCGCTC | Mutant |
| cyaBXbaIR | GCGCTCTAGA GAGTCGTCACCAGCCTGCTGG | Mutant |
| cyaBSOEF | CCTCTGGAGCGCATGAAGCCTGTCATCCTCTAAGTTCGTCGAACG | Mutant |
| cyaBSOER | CGTTCGACGAACTTAGAGGATGACAGGCTTCATGCGCTGGAGAGG | Mutant |
| cyaBintR | GATGTATTGCAGCAGGGCGTGA | SEQ |
| cyaBEMSAF1 | CCCATCCGCGCGGCTGGCCC | EMSA |
| cyaBEMSAR1 | CATGCGCTGGAGAGGATCCCTG, biotinylated | EMSA |
| pscEF |  | EMSA |
| pscEFEMSAR | GATCTTCTGCAGGATGCCTTGC | EMSA |
| cyaBTJ1F | GCGCGAATTC G AAGCCTACCCTCCCCGACCG | Complementation |
| cyaBTJ1R | GCGCAAGCTTAGAGGATGACCTTGTCGCG | Complementation |
| fimUHindIIIF |  | EMSA |
| fimUEMSAR1 | TGACATGCGGTTGCTCCAGG | EMSA |
| pilY1Rcheck | GAAAGACACCCTGAAGCTCGAG | SEQ |
| pilY1intR2 | GTGAATGTAGATATCGGTGATG | SEQ |
| 4781GSR1 | GTCCAGCATGCTCTCCATCGC | EMSA |
| PA4781TFFHindIII | GCGCAAGCTTCTGTTCGCCGGCGAGCGGATCG | Transcriptional Fusion |
| PA4781TFRBamHI | GCGCGGATCCGTCCAGCATGCTCTCCATCGCC | Transcriptional Fusion |
| algZH175AF | GCATTCCTGTTCAACAGCCTGAACAG | SDM Mutant |
| algZH175AR | CGGGCGAATCCGCGCCTGCAG | SDM Mutant |
| algZSDMcheckR | GCTGGATGCCATAGATAAGCG | SEQ |
| cyaBEMSAR1 | CATGCGCTGGAGAGGATCCCTG | EMSA |
| cyaBEMSAF1 | CCCATCCGCGCGGCTGGCCC | EMSA |
| RBSSDMcyaBF | GAATTCGTCCCGGATCGGCGCGCTTTCC | SDM Mutant |
| RBSSDMcyaBR2 | TCGTCCCGGGGGAGCGACC | SDM Mutant |
| pilJXbaIR | GCGCTCTAGA GAGCGACGTAGTCGTGCCGG | Mutant |
| cyaAintR | CAGGCGGAATTGCTGGATGAAG | SEQ |
| pilY1intR2 | GTGAATGTAGATATCGGTGATG | SEQ |
| pilY1SOER | AGTGGTGTCGGTTCTTTCCTTCGATGGGGCG | Mutant |
| cdrATFRBamHI | GCGCGGATCCGATCGGCACCTTGTTGCTGATCG | Transcriptional Fusion |
| cdrATFFEcoRI | GCGCGAATTCATGAAAATCTCCCTATCTGCGTGG | Transcriptional Fusion |
| pHERDSF | ATCGCAACTCTCTACTGTTTCTCC | SEQ |

Table S3. Statistics for AUC comparisons of *lux* data.

| ***lacP1-lux*** |  | P value |
| --- | --- | --- |
| PAO1 vs ∆*pilY1* |  | 0.0001 |
| \| PAO1 vs. ∆*pilA* \| \| --- \| |  | 0.0001 |
| \| PAO1 vs. ∆*pilJ* \| \| --- \| |  | 0.0001 |
| PAO1 vs. ∆*cpdA* |  | 0.0001 |
| \| PAO1 vs. ∆*vfr* \| \| --- \| |  | 0.0001 |
| ***cdrA-lux*** |  |  |
| \| PAO1 vs. ∆*pilY1* \| \| --- \| |  | 0.0001 |
| \| PAO1 vs. ∆*pilA* \| \| --- \| |  | \| \| 0.0739 (NS) \| \| --- \| \| \| --- \| --- \| |
| PAO1 vs. ∆*pilJ* |  | \| 0.6034 (NS) \| \| --- \| |
| PAO1 vs. ∆*gacA* |  | 0.0001 |
| ***cyaB-lux*** |  |  |
| \| PAO1 vs. ∆*pilY1* \| \| --- \| |  | 0.0001 |
| \| PAO1 vs. ∆*algR* \| \| --- \| |  | \| 0.0090 \| \| --- \| |
| \| PAO1 vs. ∆*pilY1*RD54A \| \| --- \| |  | \| 0.0872 (NS) \| \| --- \| |
| \| PAO1 vs. ∆*vfr* \| \| --- \| |  | 0.0001 |
| \| ∆pilY1 vs. ∆*pilY1*RD54A \| \| --- \| |  | 0.0001 |
| ***PA4781-lux*** |  |  |
| \| PAO1 vs. ∆*algR* \| \| --- \| |  | \| 0.0006 \| \| --- \| |
| \| PAO1 vs. ∆*pilY1* \| \| --- \| |  | 0.0001 |
| \| PAO1 vs. ∆*pilY1*RD54A \| \| --- \| |  | 0.0001 |
| \| PAO1 vs. ∆*vfr* \| \| --- \| |  | \| 0.9290 \| \| --- \| |
| \| \| *∆pilY1 vs. ∆pilY1∆vfr* \| \| --- \| \| \| --- \| --- \| |  | \| 0.8661 (NS) \| \| --- \| |
| ***Z-lux*** |  |  |
| \| PAO1 vs. ∆*algR* \| \| --- \| |  | 0.0001 |
| \| PAO1 vs. ∆*pilY1* \| \| --- \| |  | 0.0001 |
| \| PAO1 vs. ∆*pilY1*RD54A \| \| --- \| |  | 0.0001 |
| ***R-lux*** |  |  |
| \| PAO1 vs. ∆*algU* \| \| --- \| |  | 0.0001 |
| \| PAO1 vs. ∆*pilY1* \| \| --- \| |  | \| 0.6647 (NS) \| \| --- \| |
| \| PAO1 vs. ∆*pilY1*RD54A \| \| --- \| |  | 0.0001 |
|  |  |  |


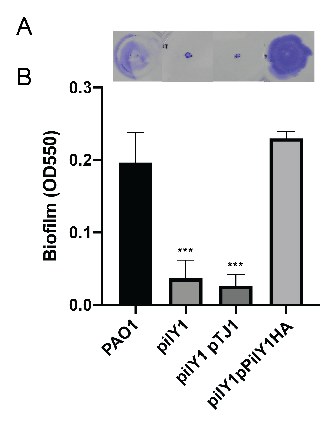


Figure S1. Complemetation of ∆*pilY1*. Single copy complementation on the chromosome with *pilY1HA*.A. twiching motility assay. B. Restoration of biofilm formation. pTJ1 is the integrating vector used as a control. All media used were supplemented with 0.1% arabinose for induction. Significance was determined using a one-way ANOVA with Tukey’s post-test (n=3). ***, *P*<0.001


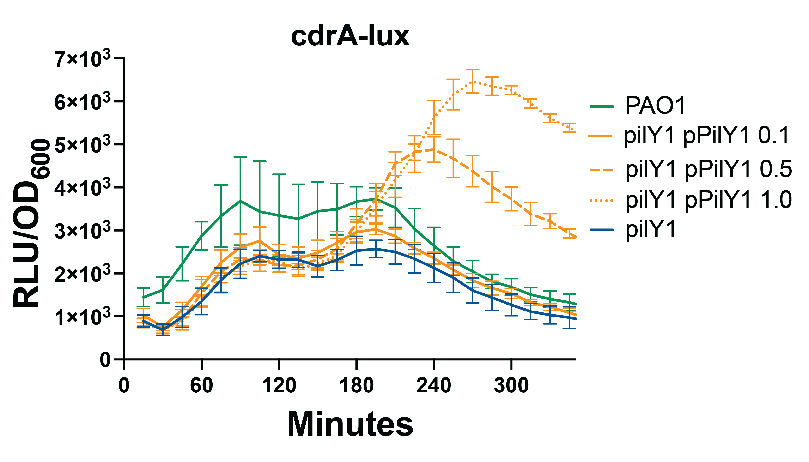


Figure S2. Complementation of ∆*pilY1* with pPilY1 containing the *cdrA-lux* transcriptional fusion. Increasing arabinose concentrations are indicated in the legend. n=3

Figure S3. Transcriptional analysis of cAMP components to determine increased cAMP in the ∆*pilY1* strain. A. *cyaA-lux* represents expression of one of the adenylate cyclases. B. Transcriptional fusion of cpdA-lux representing the cAMP phosphodiesterase.n=3


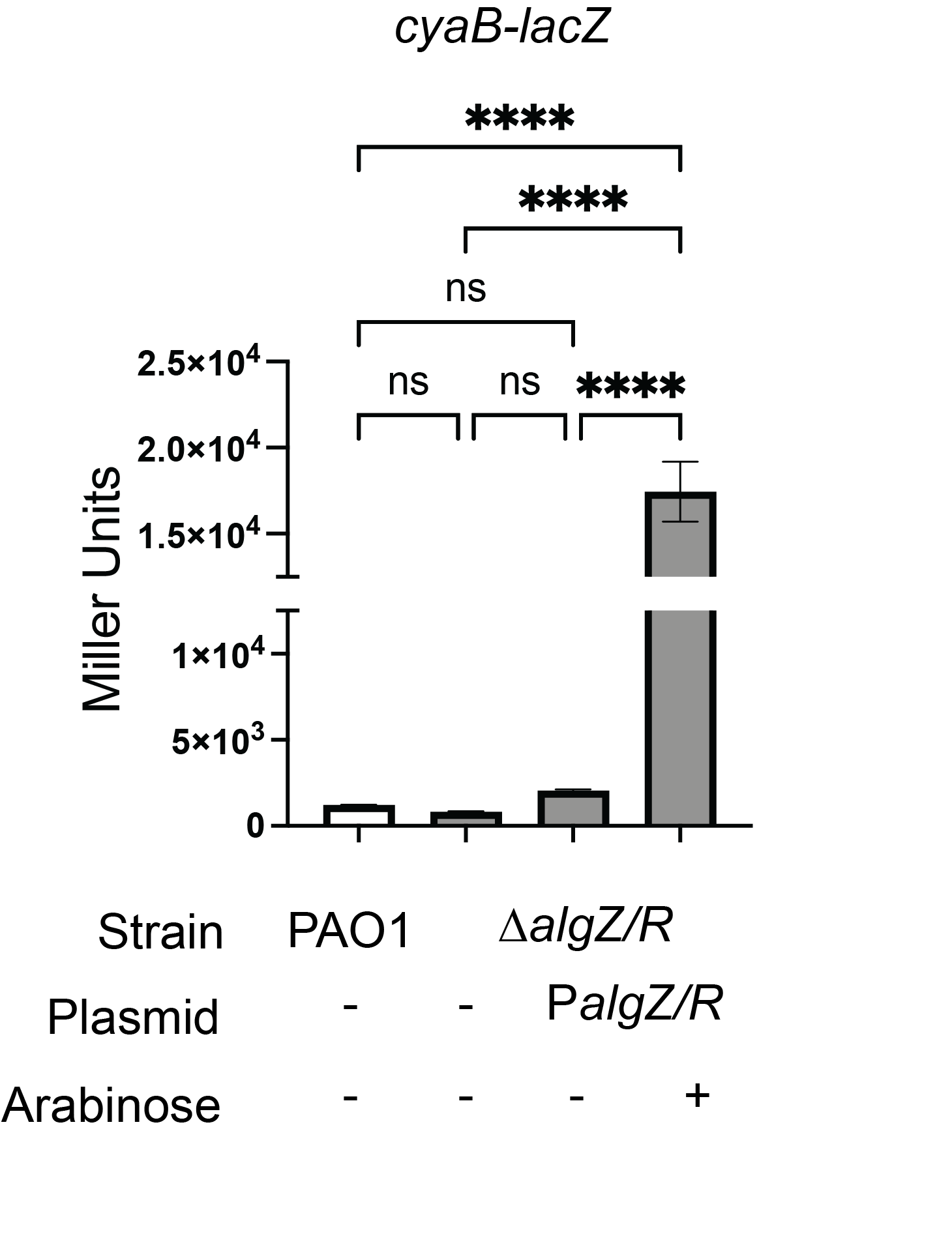


Figure S4. Overexpression of *algZ/R* increases *cyaB-lacZ* reporter activity. Significance was determined using a one-way ANOVA and Tukey’s post-test. ****, *P*<0.0001

References

1. **Miller VL, Mekalanos JJ.** 1988. A novel suicide vector and its use in construction of insertion mutations: osmoregulation of outer membrane proteins and virulence determinants in Vibrio cholerae requires toxR. J Bacteriol **170:**2575-2583.

2. **Figurski DH, Helinski DR.** 1979. Replication of an origin-containing derivative of plasmid RK2 dependent on a plasmid function provided *in trans*. Proc Natl Acad Sci USA **76:**1648-1652.

3. **Hoang TT, Karkhoff-Schweizer RR, Kutchma AJ, Schweizer HP.** 1998. A broad-host-range Flp-FRT recombination system for site-specific excision of chromosomally-located DNA sequences: application for isolation of unmarked *Pseudomonas aeruginosa* mutants. Gene **212:**77-86.

4. **Holloway BW.** 1955. Genetic recombination in *Pseudomonas aeruginosa*. J Gen Microbiol **13:**572-581.

5. **Holloway BW, Krishnapillai V, Morgan AF.** 1979. Chromosomal genetics of *Pseudomonas*. Microbiol Rev **43:**73-102.

6. **Mathee K, Ciofu O, Sternberg C, Lindum PW, Campbell JI, Jensen P, Johnsen AH, Givskov M, Ohman DE, Molin S, Hoiby N, Kharazmi A.** 1999. Mucoid conversion of Pseudomonas aeruginosa by hydrogen peroxide: a mechanism for virulence activation in the cystic fibrosis lung. Microbiology **145 ( Pt 6):**1349-1357.

7. **Hanna SL, Sherman NE, Kinter MT, Goldberg JB.** 2000. Comparison of proteins expressed by Pseudomonas aeruginosa strains representing initial and chronic isolates from a cystic fibrosis patient: an analysis by 2-D gel electrophoresis and capillary column liquid chromatography-tandem mass spectrometry. Microbiology **146 ( Pt 10):**2495-2508.

8. **Stacey SD, Pritchett CL.** 2016. Pseudomonas aeruginosa AlgU Contributes to Posttranscriptional Activity by Increasing rsmA Expression in a mucA22 Strain. J Bacteriol **198:**1812-1826.

9. **Schuster M, Lostroh CP, Ogi T, Greenberg EP.** 2003. Identification, timing, and signal specificity of Pseudomonas aeruginosa quorum-controlled genes: a transcriptome analysis. J Bacteriol **185:**2066-2079.

10. **Liu PV.** 1973. Exotoxins of *Pseudomonas aeruginosa*. I. Factors that influence the production of exotoxin A. J Infect Dis **128:**506-513.
